# Identification of Distinct Unmutated Chronic Lymphocytic Leukemia Subsets in Mice Based on Their T Cell Dependency

**DOI:** 10.3389/fimmu.2018.01996

**Published:** 2018-09-13

**Authors:** Simar Pal Singh, Marjolein J. W. de Bruijn, Mariana P. de Almeida, Ruud W. J. Meijers, Lars Nitschke, Anton W. Langerak, Saravanan Y. Pillai, Ralph Stadhouders, Rudi W. Hendriks

**Affiliations:** ^1^Department of Pulmonary Medicine, Erasmus MC, Rotterdam, Netherlands; ^2^Department of Immunology, Erasmus MC, Rotterdam, Netherlands; ^3^Post-graduate School Molecular Medicine, Erasmus MC, Rotterdam, Netherlands; ^4^Department of Genetics, University of Erlangen, Erlangen, Germany; ^5^Department of Cell Biology, Erasmus MC, Rotterdam, Netherlands

**Keywords:** B cell receptors, T cell help, BCR signaling, Bruton's tyrosine kinase, chronic lymphocytic leukemia

## Abstract

Chronic lymphocytic leukemia (CLL) can be divided into prognostically distinct subsets with stereotyped or non-stereotyped, mutated or unmutated B cell receptors (BCRs). Individual subsets vary in antigen specificity and origin, but the impact of antigenic pressure on the CLL BCR repertoire remains unknown. Here, we employed *IgH.TE*μ mice that spontaneously develop CLL, expressing mostly unmutated BCRs of which ~35% harbor V_H_11-2/Vκ14-126 and recognize phosphatidylcholine. Proportions of V_H_11/Vκ14-expressing CLL were increased in the absence of functional germinal centers in *IgH.TE*μ mice deficient for CD40L or activation-induced cytidine deaminase. Conversely, *in vivo* T cell-dependent immunization decreased the proportions of V_H_11/Vκ14-expressing CLL. Furthermore, CLL onset was accelerated by enhanced BCR signaling in *Siglec-G*^−/−^ mice or in mice expressing constitutively active Bruton's tyrosine kinase. Transcriptional profiling revealed that V_H_11 and non-V_H_11 CLL differed in the upregulation of specific pathways implicated in cell signaling and metabolism. Interestingly, principal component analyses using the 148 differentially expressed genes revealed that V_H_11 and non-V_H_11 CLL clustered with BCR-stimulated and anti-CD40-stimulated B cells, respectively. We identified an expression signature consisting of 13 genes that were differentially expressed in a larger panel of T cell-dependent non-V_H_11 CLL compared with T cell-independent V_H_11/Vκ14 or mutated *IgH.TE*μ CLL. Parallel differences in the expression of these 13 signature genes were observed between heterogeneous and stereotypic human unmutated CLL. Our findings provide evidence for two distinct unmutated CLL subsets with a specific transcriptional signature: one is T cell-independent and B-1 cell-derived while the other arises upon antigen stimulation in the context of T-cell help.

## Introduction

Chronic lymphocytic leukemia (CLL) is the most common adult leukemia characterized by an accumulation of monoclonal CD5^+^ mature B cells with low surface immunoglobulin (Ig) expression in peripheral blood (1).

CLL is a clinically and molecularly heterogeneous disease whereby progression is influenced by many factors. One-third of patients can be classified as stereotypic CLL, in which BCRs are highly similar between patients (2). The remaining two-third of CLL either lack or have limited similarity with stereotyped CLL BCRs. This classification provides strong molecular evidence for antigen selection in CLL pathogenesis (2). CLL can also be grouped based on IGHV mutational status (3, 4). Significant (>2%) somatic hypermutation (SHM) is observed in patients with mutated CLL (M-CLL), who often develop indolent disease. SHM is absent in unmutated CLL (U-CLL) which evolves rapidly and has a less favorable prognosis (4). The SHM status provides a robust and stable prognostic marker, independently of clinical stage and other markers (5). Furthermore, it reinforces the role of selection by self-antigens or exogenous antigens in CLL pathogenesis. CLL cells show constitutive activation of several BCR downstream kinases, increasing leukemic cell survival *in vitro* (6). In support, small molecule inhibitors of BCR–associated kinases including Bruton's tyrosine kinase (Btk) have shown impressive clinical anti-tumor activity (7, 8).

Few external antigens that potentially drive CLL *in vivo* have been identified; CLL cells were shown to display antigen-independent, cell-autonomous signaling mediated by auto-recognition (9). Several reports have shown that U-CLL express polyreactive BCRs that bind with low affinity to various auto-antigens generated during apoptosis or oxidation (10, 11). In this respect, they resemble natural antibodies secreted by B-1 cells in mice. B-1 cells are a self-renewing CD5^+^ B cell population with remarkably restricted IGHV gene usage and low or no SHM (12). B-1 cells are thought to be generated based on positive selection, by virtue of their receptor specificities to self-antigens, independent of T-cell help (12). Adding to this complexity, the antigen specificity of U-CLL includes both T cell-independent (TI) and T cell-dependent (TD) antigens (11, 13, 14). On the other hand, M-CLL express BCRs that are believed to bind with high-affinity to auto-antigens and show activation of pathways associated with anergic B cells (15, 16).

Differences regarding BCR reactivity have fueled several theories concerning the cellular origins of CLL. SHM status and transcription profiling indicated that U-CLL and M-CLL are derived from CD5^+^CD27^−^ pre- and CD5^+^CD27^+^ post-germinal center (GC) B cells, respectively (17, 18). Extrafollicular or marginal zone (MZ) B cell responses, involving the activation of low-affinity B cells to TI antigens with low SHM, could also be relevant for CLL (19). Direct *in vivo* evidence for the TD or TI origin of CLL subgroups is still missing, mainly due to a lack of mouse models that spontaneously develop both stereotypic and non-stereotypic, mutated and unmutated CLL (20). In the widely studied *E*μ*-TCL1* model, CLL predominantly express unmutated stereotyped *IghV11* or *IghV12* BCRs (21). The *IgH.TE*μ CLL mouse model that we previously generated is based on sporadic expression of the SV40 large T oncogene in mature B cells (22). This was achieved by SV40 large T insertion in opposite transcriptional orientation into the *IgH* locus D_H_-J_H_ region. In contrast to the *E*μ*-TCL1* model, *IgH.TE*μ mice mainly develop unmutated CLL with a diverse *IghV* repertoire, and at low frequencies mutated CLL (20, 22). Because of their mixed sv129xC57BL/6 background, we used IgMa/IgMb allotype expression to define CLL incidence by the accumulation of >70% IgMb^+^ B-cells (22, 23). Aging *IgH.TE*μ mice show accumulation of monoclonal CLL-like CD5^+^CD43^+^IgM^+^IgD^low^CD19^+^ B cells around nine months of age. Although constitutive Btk signaling was not apparent in primary *IgH.TE*μ CLL cells, CLL development was dependent on Btk. Btk-mediated signaling enhanced leukemogenesis and Btk-deficiency led to a complete rescue from the disease (23). Moreover, primary CLL cells from *IgH.TE*μ mice or stable cell lines generated from these mice had detectable expression of p-Akt and substantial levels of p-S6, both of which function downstream of the BCR (23, 24).

To address the impact of antigenic pressure on BCR selection in CLL, we analyzed the effects of defective T cell help and GC formation, as well as robust antigenic stimulation on CLL development in *IgH.TE*μ mice. We show that there are two distinct unmutated CLL subsets present in the *IgH.TE*μ mouse model. The V_H_11-2/Vκ14-126-expressing CLL developed independently of T-cell help. Conversely, non-V_H_11 CLL was TD and displayed a specific transcriptional signature associated with non-stereotypic U-CLL in human. These findings provide evidence for differential dependence on T cell help in unmutated CLL in mice and suggest that development of human U-CLL can also be T cell-dependent.

## Materials and methods

### Mice

Mice (C57BL/6) deficient for *Cd40l* (25), *Aicda* (26) *or Siglec-G* (27), and *Cd19-E-Btk-2* (28) transgenic mice were crossed to *IgH.TE*μ mice (F1 sv129xC57BL/6). CLL development was monitored every 3–6 weeks by screening peripheral blood for a monoclonal B cell expansion using flow cytometry. CLL formation was defined by accumulation of >70% IgMb^+^ B-cells in the peripheral blood of the mice. Mice were sacrificed after detection of CLL. Mice were bred and kept in the Erasmus MC experimental animal facility and experiments were approved by the Erasmus MC committee of animal experiments.

### Patients and healthy controls

Primary patient material was obtained from peripheral blood from CLL patients, while peripheral blood from healthy controls (>50 years of age) was obtained via Erasmus MC and via Sanquin blood bank (Rotterdam). Diagnostic and control samples were collected upon informed consent and anonymized for further use, following the guidelines of the Institutional Review Board, and in accordance with the declaration of Helsinki. The BCR characteristics of all CLL patients are included in Supplementary Table 5. Peripheral blood mononuclear cells (PBMCs) were isolated using Ficoll Hypaque (GE Healthcare, Little Chalfont, UK) according to the manufacturer's instructions. Naïve mature B cells were isolated from healthy control PBMCs using FACS-purification for CD19+CD27-IgD+ cells. The purity of naïve mature healthy B cell samples was >95% as determined by flow cytometry.

### *In vivo* immunizations

TD immune responses were induced by i.p. immunization. Primary immunizations were induced in 10-12-week-old mice with 100 μg TNP-KLH on alum. After 5 weeks this was followed by a secondary immunization with 100 μg TNP-KLH in PBS (28).

### BCR sequencing

Primer sequences and PCR condition were previously described (22, 23). PCR products were directly sequenced using the BigDye terminator cycle sequencing kit with AmpliTaq DNA polymerase on an ABI 3130xl automated sequencer (Applied Biosystems). Sequences were analyzed using IMGT/V-Quest (http://www.imgt.org, using Ig gene nomenclature as provided by IMGT). All sequences were confirmed in at least one duplicate analysis.

### Flow cytometry procedure

Preparation of single-cell suspensions of lymphoid organs and lysis of red blood cells were performed according to standard procedures. Cells were (in)directly stained in flow cytometry buffer (PBS, supplemented with 0.25% BSA, 0.5 mM EDTA and 0.05% sodium azide) using the following fluorochrome or biotin-conjugated monoclonal antibodies or reagents: anti-B220 (RA3-6B2), anti-CD19 (ID3), anti-CD5 (53-7.3), anti-CD43 (R2/60), anti-CD23 (B3B4) all from eBioscience and anti-CD138 (281-2), anti-CD95 (Jo2), anti-IgD (11-26), anti-IgMb (AF6-78), anti-IgMa (DS-1), anti-Igλ (R26-46), anti-Igκ (187.1), anti-CD21 (7G6), all from BD biosciences, using conjugated streptavidin (eBioscience) as a second step for biotin-conjugated antibodies.

Leukemic cells (CD19^+^CD5^+^) were stained with fluorescein-labeled phosphatidylcholine (PtC) liposomes (DOPC/CHOL 55:45, Formumax Scientific Inc.) in flow cytometry buffer. Cells were co-stained with anti-CD19, anti-CD43, or anti-CD5 (BD Biosciences).

### MACS cell sorting

Splenic single-cell suspensions were prepared in magnetic-activated cell sorting (MACS) buffer (PBS/2mM EDTA/0.5%BSA) and naïve splenic B cells from 8–12 week-old WT C57BL/6 mice were purified by MACS, as previously described (24, 29). Non-B cells, B-1 cells, GC B cells, and plasma cells were first labeled with biotinylated antibodies (BD Biosciences) to CD5 (53–7.3), CD11b (M1-70), CD43 (S7), CD95 (Jo2), CD138 (281-2), Gr-1 (RB6-8C5), and TER-119 (PK136) and subsequently with streptavidin-conjugated magnetic beads (Miltenyi Biotec). Purity of MACS-sorted naïve B cells was confirmed by flow cytometry (typically > 99% CD19+ cells). To obtain activated B cells, purified naïve WT B cells were cultured in culture medium [RPMI 1640 (life technologies)/10% FCS (gibco)/50 μg/mL gentamycin(life technologies)/0,05 mM ß-mercaptoethanol (Sigma)] in the presence of 10 μg/ml F(ab')2 anti-IgM (Jackson Immunoresearch) for 12 h.

### RNA-sequencing

RNA was extracted from naive or activated splenic B cells, as well as from purified (using MACS-purification for CD19+ cells) primary tumors from IgH.TEμ mice with the RNeasy Micro kit (Qiagen) according to manufacturer's instructions. The TruSeq RNA Library Prep kit (Illumina) was used to construct mRNA sequencing libraries that were sequenced on an Illumina HiSeq 2500 (single read mode, 36 bp read length). Raw reads were aligned using Bowtie to murine transcripts (RefSeq database) from the University of California at Santa Cruz (UCSC) mouse genome annotation (NCBI37/mm9) (30). Differential gene expression analysis was performed using DESeq2 (31) with an adjusted *P*-value (false discovery rate; FDR) of *P* < 0.05. Log2-fold changes and FDR values as calculated by DESeq2 were used to generate a volcano plot using R (R studio version 1.1.383). Normalized gene expression levels quantified as reads per kilobase of a transcript per million mapped reads (RPKMs) were used for various clustering approaches (unsupervised hierarchical clustering, supervised clustering, and PCA) that were performed using R and PAST software (https://folk.uio.no/ohammer/past/). Visualization of clustering analysis output was performed using R, PAST, and Java TreeView (32). Molecular pathway enrichments were obtained from the online MSigDB database. Gene expression data for anti-CD40 plus IL-4 stimulated follicular B-cells was obtained from previously reported data and downloaded from the Gene Expression Omnibus (GEO; accession number GSE77744) (33). RNA-Seq data generated in this study have been deposited in the GEO database (accession number GSE117713).

### Quantitative real time PCR analysis

Samples tested in qRT-PCR were from *IgH.TE*μ (7 V_H_11 and 15 non-V_H_11), from *IgH.TE*μ*.Aicda*^−/−^ (4 V_H_11 and 4 non-V_H_11), and from *IgH.TE*μ*.TD* (4 non-V_H_11) mouse groups. For quantitative RT-PCR analysis, TaqMan probes were employed. Probe Finder software (Roche Applied Science), the Universal Probe Library (Roche Applied Science) and Ensembl genome browser (http://www.ensembl.org/) were used for primer and probe design. Taqman Universal Master Mix II, was purchased from Thermo Fisher Scientific. Quantitative RT-PCR was performed by using the 7300 Real Time PCR system (Applied Biosciences) according to manufacturer's instructions. Gene expression was analyzed with an ABI Prism 7300 Sequence Detector and ABI Prism Sequence Detection Software version 1.4 (Applied Biosystems). Cycle-threshold levels were calculated for each gene and the housekeeping gene glyceraldehyde-3-phosphate dehydrogenase (*Gapdh*) was used for normalization of the values. All primer sequences and probe numbers are listed in Supplementary Table 7.

### Statistical analysis

Statistical analysis was performed using GraphPad Prism software (San Diego, California, USA) or R. The log rank test was used for calculating the level of significance for survival differences between mouse groups. The Chi-square test was used to determine the significance for BCR usage differences between different mouse groups. To evaluate differences in expression levels of different genes by qRT-PCR we used a Mann-Whitney *U*-test between two groups or a Kruskal-Wallis test corrected with Dunn's multiple comparison test for more than two groups.

## Results

### Two subsets of unmutated CLL arise in *IgH.TEμ* mice

**Figure 1:**
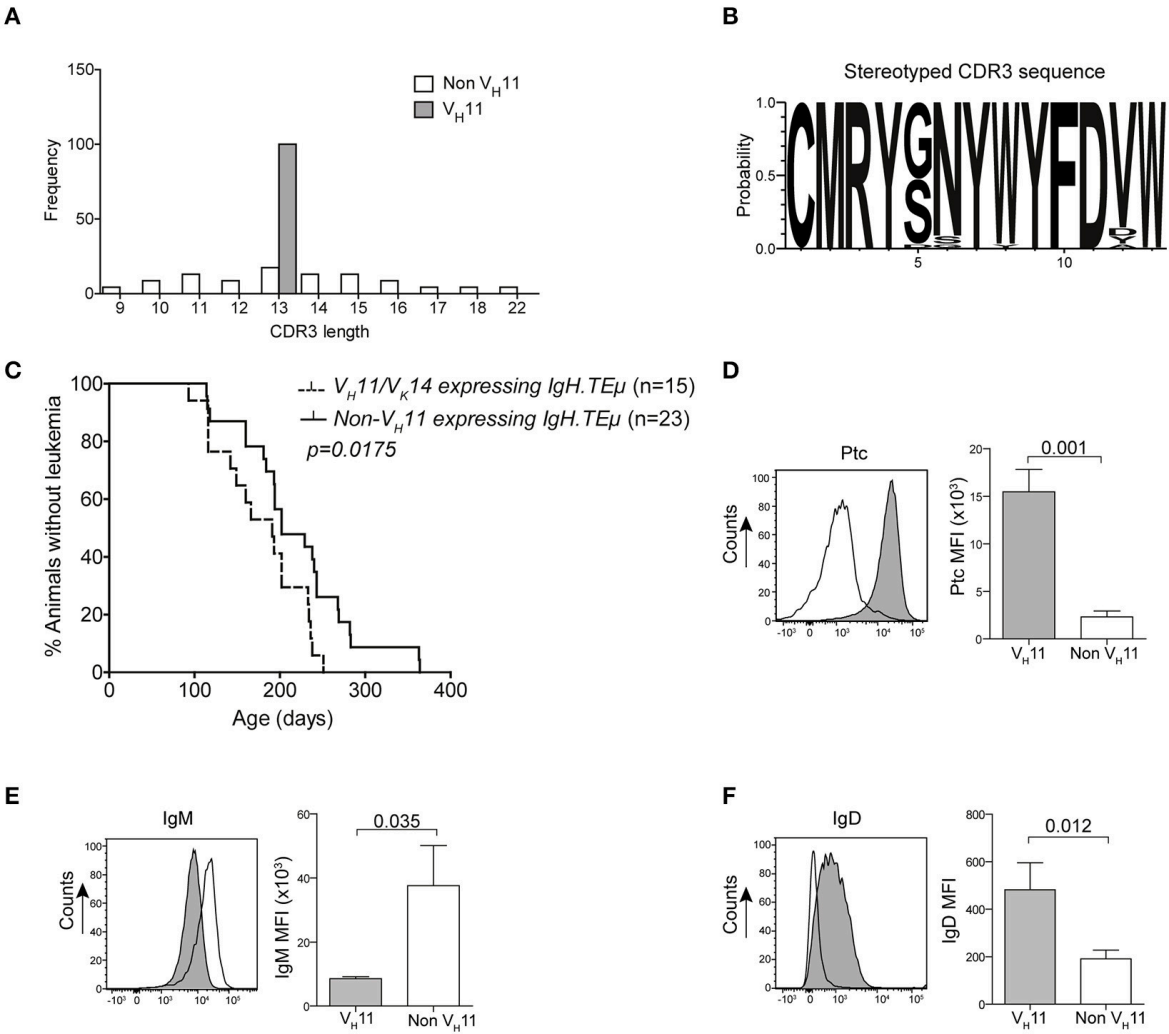
Early onset of disease in VH11 expressing CLL from *IgH.TE*μ mice. **(A)** Bar graphs summarizing the distribution of CDR3 length in V_H_11 (*gray, n* = 15) vs. non-V_H_11 (*white, n* = 23) CLL from *IgH.TE*μ mice. **(B)** Web logo depicting stereotyped CDR3 amino acid sequence of V_H_11 (*n* = 15) CLL from *IgH.TE*μ mice. **(C)** Retrospective Kaplan-Meier incidence curves including *IgH.TE*μ mice with identified V_H_11 (*dotted line*) or non-V_H_11 BCR CLL (*solid line*). **(D–F)** Histogram showing flow cytometric analysis of CD19^+^CD5^+^CD43^+^ splenic CLL cells from V_H_11 (*gray*) vs. non-V_H_11 (*black line*) CLL from *IgH.TE*μ mice, stained with **(D)** phosphatidylcholine (PtC) liposomes or fluorochrome conjugated **(E)** anti-IgM or **(F)** anti-IgD antibodies. Bar graphs summarize mean fluorescence intensity of V_H_11 (*gray*) and non-V_H_11 CLL (*n* = 6 per group).

To analyze the BCR repertoire, we aged a panel of *IgH.TE*μ mice and collected blood every 3–6 weeks to monitor CLL incidence. Hereby, CLL incidence was defined by the accumulation of >70% IgMb^+^ B-cells, which displayed a CLL-like CD5^+^CD43^+^IgM^+^IgD^low^CD19^+^ phenotype (22, 23). We performed sequencing analyses of Ig heavy (*Igh*) and light (*Igl*) chain transcripts and found that a substantial proportion (~36%) of CLL in *IgH.TE*μ mice expressed stereotyped BCRs consisting of the V_H_11-2 *Igh* chain, with similar *Igh* CDR3 length and amino acid sequences, and the Vκ14-126 *Igl* chain (22, 23) (Supplementary Table 1, Figures 1A,B). The V_H_11/Vκ14 CLL mice exhibited an earlier disease onset compared with *IgH.TE*μ mice with non-stereotypic (non-V_H_11) BCR (mean incidence age 184 days and 219 days, respectively, *p* = 0.0175) (Figure 1C). In wild-type mice the V_H_11-2/Vκ14-126 BCR is preferentially expressed by B-1 lymphocytes and shows specificity to phosphatidylcholine (PtC) (12). We could confirm PtC-binding specificity of V_H_11-2 BCRs on CLL cells (Figure 1D). V_H_11 CLL showed decreased surface IgM expression and increased surface IgD expression compared to non-V_H_11 CLL (Figures 1E,F). A major proportion (~65%) of the remaining non-V_H_11 CLL expressed a J558 V_H_1-family BCR with heterogeneous CDR3 length, amino acid sequence and *Igl* chain usage (Supplementary Table 1). V_H_1 CLL showed delayed disease onset (mean incidence age 231 days), compared with V_H_11 CLL (Supplementary Figure 1).

In conclusion, based on *Ig* gene usage we could distinguish different subsets of unmutated *IgH.TE*μ CLL displaying differential disease onset.

### Germinal center defects lead to increased V_H_11/Vκ14 usage in unmutated CLL

**Figure 2:**
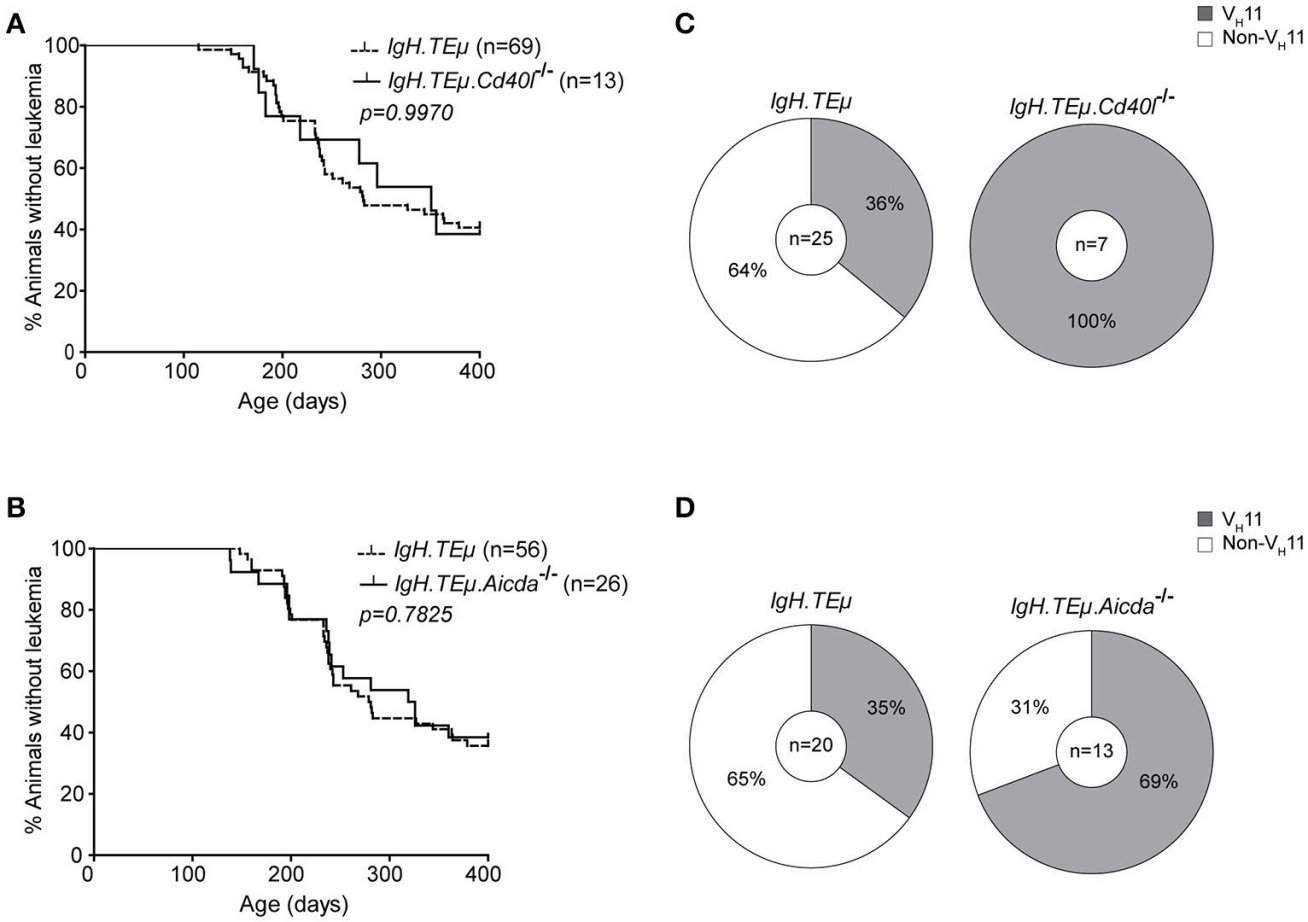
Increased frequency of V_H_11 usage in CLL from germinal center attenuated *IgH.TE*μ mice. **(A,B)** Kaplan-Meier incidence curves of **(A)** *IgH.TE*μ (*dotted line*) vs. *IgH.TE*μ*.Cd40l*^−/−^ (*solid line*) mice or **(B)** *IgH.TE*μ (*dotted line*) vs. *IgH.TE*μ*.Aicda*^−/−^ (*solid line*) mice. **(C,D)** Pie charts summarizing frequencies of V_H_11 BCR (*gray*) and non- V_H_11 BCR-expressing CLL in the indicated mouse groups.

Because V_H_11/Vκ14-expressing CLL likely originate from B-1 cells, we hypothesized that they should still develop in the absence of functional GCs. Therefore, we investigated their dependence on functional GCs and T cell help by crossing *IgH.TE*μ mice with *Cd40l*^−/−^ or *Aicda*^−/−^ mice. *Cd40l* ^−/−^ or *Aicda*^−/−^ mice display a complete lack or aberrant enlargement of GCs, respectively, paralleling the human hyper-IgM syndrome phenotype (26, 34). We monitored CLL incidence, as described above, in cohorts of *Cd40l*-deficient *IgH.TE*μ mice (*IgH.TE*μ*.Cd40l*^−/−^, *n* = 13), *Aicda*-deficient *IgH.TE*μ mice (*IgH.TE*μ*.Aicda*^−/−^, *n* = 26) and *IgH.TE*μ control littermates, *n* = 69 or *n* = 56, respectively for ~400 days (Figure 2). CLL frequency and onset was not altered in *IgH.TE*μ*.Cd40l*^−/−^mice (~59%, compared with ~62% in *IgH.TE*μ control littermates; *p* = 0.99) or in *IgH.TE*μ*.Aicda*^−/−^ mice (~62%, compared with ~64% in *IgH.TE*μ control littermates; *p* = 0.78) (Figures 2A,B).

To explore the impact of CD40L or AID-deficiency on BCR usage in CLL, we performed *IghV* and *IglV* sequence analyses in selected CLL samples with high tumor load (>95% IgMb^+^CD5^+^CD43^+^CD19^+^ CLL-like cells) (Supplementary Table 1). Interestingly, usage of the stereotypic V_H_11/Vκ14 BCR was significantly increased in CLL from *IgH.TE*μ*.Cd40l*^−/−^ mice (*n* = 7/7, 100%), compared with control *IgH.TE*μ mice (*n* = 9/25, ~36%, Chi-square *p* < 0.001). Also CLL from *IgH.TE*μ*.Aicda*^−/−^ mice showed increased V_H_11/Vκ14 usage (*n* = 9/13, ~69%) compared with control littermates (*n* = 7/20, ~35%, Chi-square *p* < 0.01) (Figures 2C,D). These V_H_11 CLL also expressed similar *Igh* CDR3 sequences (Supplementary Table 1).

Taken together, these findings indicate that V_H_11/Vκ14-expressing CLL arise independently of T cell help or GC formation, whereas non-V_H_11 CLL is T cell-dependent and reduced in the absence of functional GCs in *IgH.TE*μ*.Cd40l*^−/−^and *IgH.TE*μ*.Aicda*^−/−^ mice.

### T-cell dependent antigenic stimulation of B cells *in vivo* reduces V_H_11/Vκ14 usage in unmutated *IgH.TEμ* CLL

**Figure 3:**
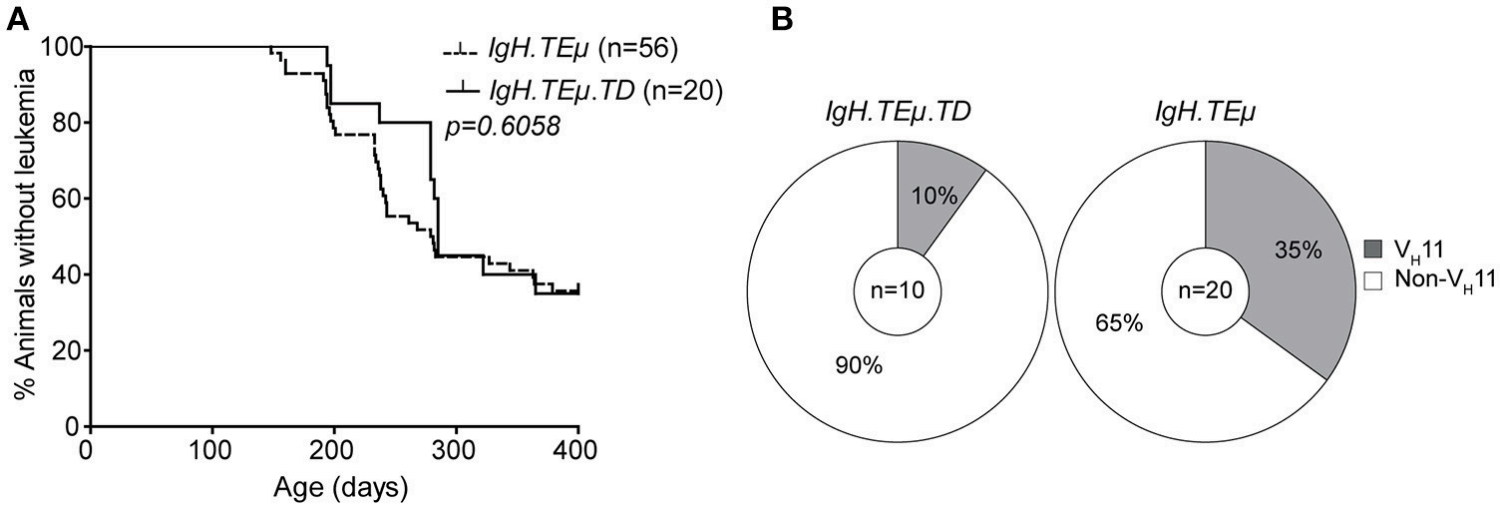
CLL V_H_11 usage is dependent on antigenic stimulation. **(A)** Kaplan-Meier incidence curves of *IgH.TE*μ (*dotted line*) vs. *IgH.TE*μ*.TD* (*solid line*). **(B)** Pie charts summarizing the frequencies of V_H_11 (*gray*) and non-V_H_11 BCR-expressing CLL in the indicated mouse groups.

To directly investigate whether antigenic stimulation in the context of T cell help affects CLL onset and the CLL BCR repertoire, we immunized *IgH.TE*μ mice with TNP-KLH coupled to alum (*IgH.TE*μ*.TD, n* = 20) to induce a TD B cell response. CLL onset did not differ between immunized and non-immunized littermates (*n* = 56) (Figure 3A). At the age of ~400 days, CLL incidence in *IgH.TE*μ*.TD* mice was ~65% similar to non-immunized control *IgH.TE*μ mice (~62%) (Figure 3A).

Next, we analyzed *IghV* and *IglV* sequences in CLL samples with high tumor load (>95% IgMb^+^CD5^+^CD43^+^CD19^+^ CLL-like cells). In contrast to control *IgH.TE*μ mice, which showed ~35% (*n* = 7/20) V_H_11 usage, only 10% (*n* = 1/10) of *IgH.TE*μ*.TD* CLL expressed a V_H_11/Vκ14 BCR (Chi-square *p* = 0.09) (Figure 3B). The majority (*n* = 6/9, 67%) of CLL in *IgH.TE*μ*.TD* mice expressed a J558/V_H_1-family *IghV* gene and we did not observe mutated CLL (Supplementary Table 1).

In summary, we found that robust TD immunization favors development of non-V_H_11 CLL.

### Enhanced BCR signaling accelerates disease onset in *IgH.TEμ* mice

Our findings provide evidence that T cell-derived activation or selection signals, in particular CD40L, shape the BCR repertoire of CLL in *IgH.TE*μ mice, but do not significantly affect disease onset or progression. It is therefore conceivable that in the *IgH.TE*μ mouse model, BCR-derived signals may be more decisive for disease progression.

To monitor the impact of BCR signaling strength on CLL development and *IghV* gene selection, we first crossed *IgH.TE*μ mice with *E-Btk-2* transgenic mice. These mice express the constitutive active E41K-BTK mutant selectively in the B-cell lineage driven by the CD19 promoter (28). The E41K mutation enhances Btk membrane localization and thereby its activation by Syk or Src-family tyrosine kinases (35). *E-Btk-2* mice show defective follicular B cell survival and a relative expansion of splenic B-1 cells (28). Flow cytometry analysis of *E-Btk-2* B-1 cells did not reveal detectable PtC binding, indicating that V_H_11 BCR expression was limited (data not shown).

**Figure 4:**
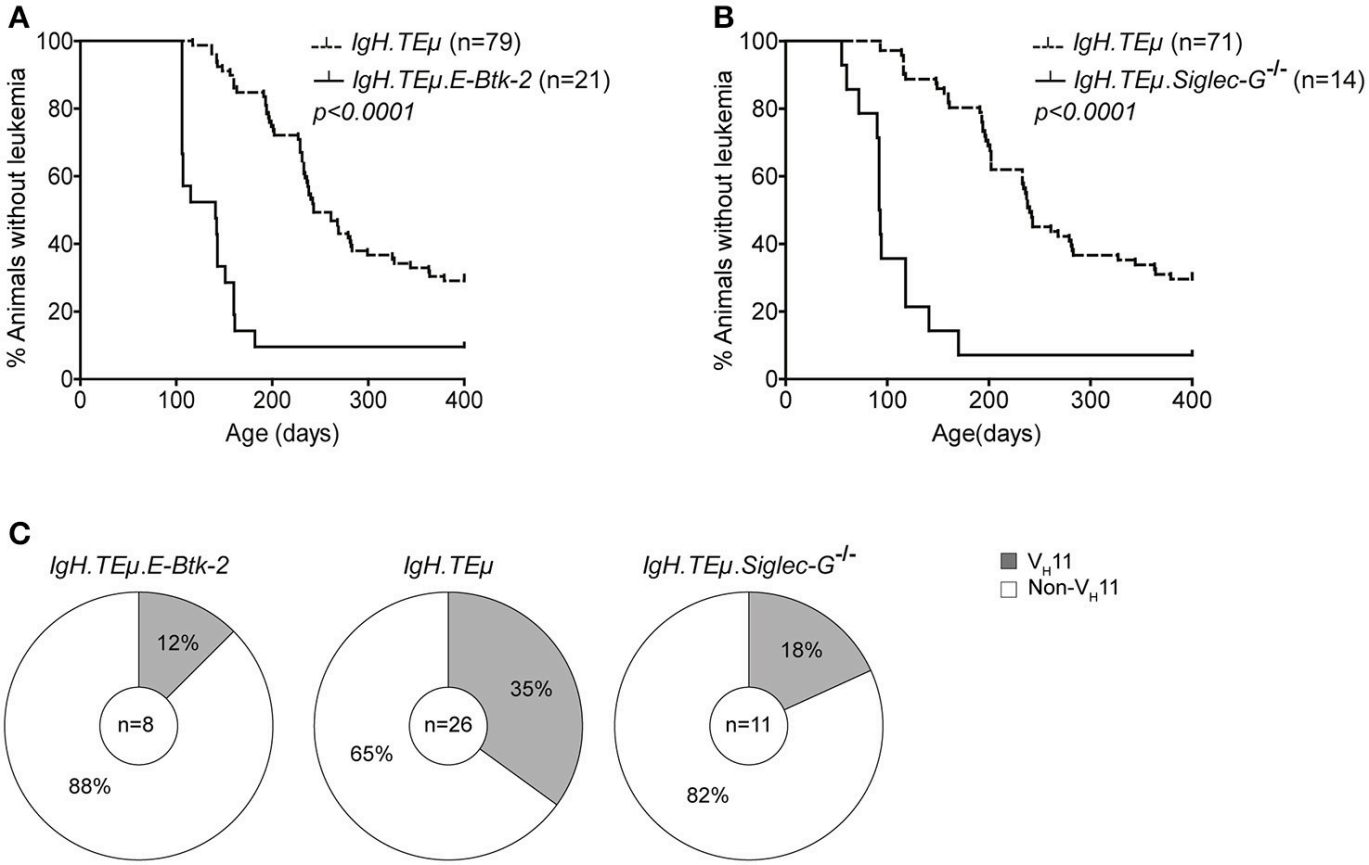
Onset of CLL is dependent on BCR signaling capacity in *IgH.TE*μ mice. Kaplan-Meier incidence curves of **(A)** *IgH.TE*μ (*dotted line*) vs. *IgH.TE*μ*.E-Btk-2* (*solid line*) mice or **(B)** *IgH.TE*μ (dotted line) vs. *IgH.TE*μ*.SiglecG*^−/−^ (*solid line*) transgenic mice. **(C)** Pie charts summarizing the frequencies of V_H_11 (*gray*) and non-V_H_11 BCR-expressing CLL in the indicated mouse groups.

We found that *IgH.TE*μ*.E-Btk-2* mice (*n* = 21) developed CLL significantly earlier (mean age of onset of ~155 days), compared with control *IgH.TE*μ mice (~279 days; *p* < 0.0001) (Figure 4A). In addition, *IgH.TE*μ*.E-Btk-2* mice appeared to have an increased disease frequency (~90% at ~400 days, compared with ~71% for control *IgH.TE*μ mice). Sequence analysis of *Igh* revealed that 1 out of 8 (~12%) tumors from *IgH.TE*μ*.E-Btk-2* mice expressed a V_H_11 BCR, compared with 35% (*n* = 9/26) in the control *IgH.TE*μ group (Figure 4C, Supplementary Table 1). This difference was not statistically significant, but the finding of a PtC-reactive V_H_11 CLL was surprising, since PtC-binding B-1 cells were not detectable in *E-Btk-2* mice. The majority (~71%; *n* = 5/7) of the non-V_H_11 BCRs expressed J558/V_H_1-family *IghV* genes.

To confirm that enhanced BCR signaling affects disease onset, we crossed *IgH.TE*μ mice on a *Siglec-G* deficient background (*IgH.TE*μ.*Siglec-G*^−/−^). *Siglec-G* is a negative regulator of BCR-mediated signaling that is expressed in all B cells (27). It is a potent inhibitor of BCR-induced Ca^2+^ signaling and a key regulator of survival and selection of B-1 cells (36). In addition, Siglec-G-deficiency abrogates V_H_11 usage in B-1 cells (36).

Similar to *IgH.TE*μ*.E-Btk-2* mice, also *IgH.TE*μ.*Siglec-G*^−/−^ mice displayed an increased disease frequency (~93 % at ~400 days, compared with ~70% for IgH.TEμ mice), with significantly accelerated CLL onset (~121 days compared with ~268 days for *IgH.TE*μ mice; *p* < 0.0001) (Figure 4B). *IghV* analyses showed a V_H_11/Vκ14 usage of ~18% (*n* = 2/11) in *IgH.TE*μ*.Siglec-G*^−/−^ CLL vs. ~35% (9/26) in the control group (Chi-square *p* < 0.242) (Figure 4C, Supplementary Table 1). Only 2/11 *IgH.TE*μ*.Siglec-G*^−/−^ CLL expressed J558/V_H_1-family *IghV* genes.

Thus, BCR signaling strength plays an important role in CLL development in *IgH.TE*μ mice, whereby enhanced signaling accelerates disease onset. Because *E-Btk-2* or *Siglec-G*^−/−^ B-1 cells do not show detectable PtC expression, our findings suggest that those few V_H_11 B cells present are efficiently transformed to CLL in *IgH.TE*μ*.E-Btk-2* or *IgH.TE*μ*.Siglec-G*^−/−^ mice. Thus, BCR signaling strength may also affect the BCR repertoire in CLL.

### Transcriptome profiling identifies unique genes and pathway aberrations for V_H_11/Vκ14 and non-V_H_11 CLL *IgH.TEμ* mice

To further explore the biological phenotype of the V_H_11 and non-V_H_11 CLL subsets, we performed genome-wide gene expression profiling on primary *IgH.TE*μ CLL (tumor load >95%) expressing either a V_H_11 (*n* = 3) or a non-V_H_11 (*n* = 3) BCR. As a reference we included resting unstimulated (un-B, *n* = 4) and anti-IgM stimulated (αIgM-B, *n* = 4) naïve splenic B cells from wild-type mice. Normalized gene expression values (see Methods for details) were used for principle component analysis (PCA). The first two principal components, which represented ~70% of the total variation among the different samples analyzed, identified three separate clusters, corresponding to un-B, αIgM-B and primary *IgH.TE*μ CLL samples, indicating a strong correlation between biological replicates (Supplementary Figure 2).

**Figure 5:**
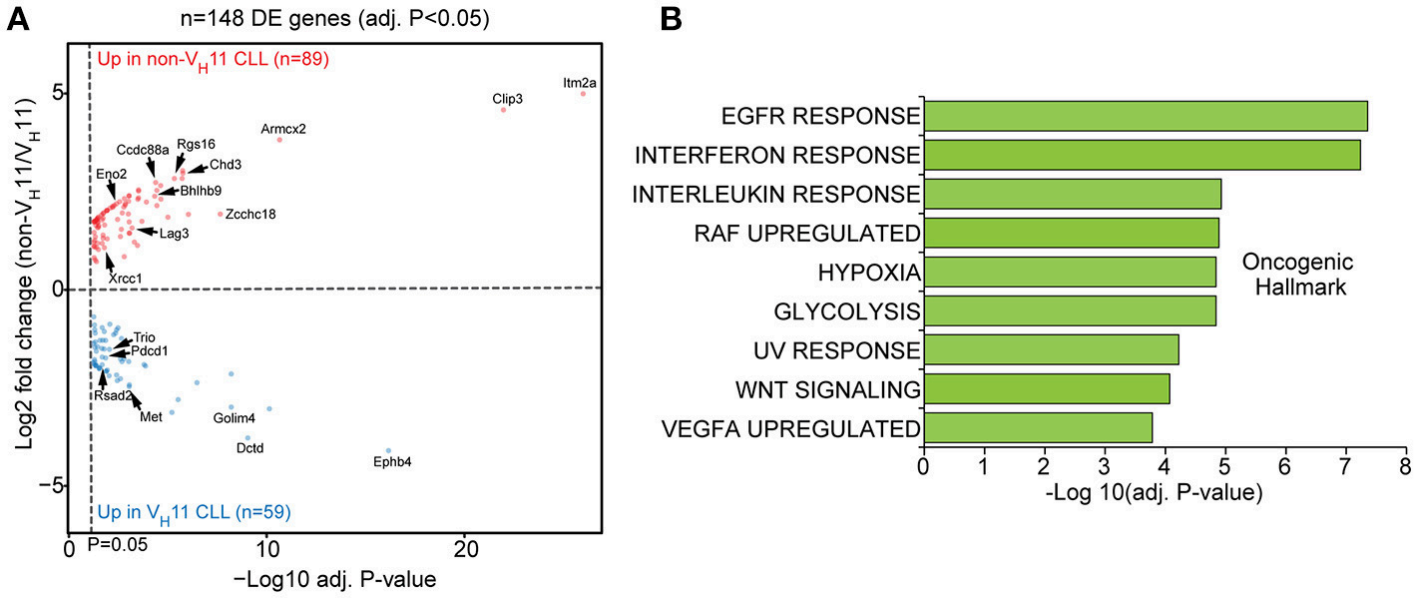
A unique set of genes and pathways is upregulated in V_H_11 vs. non-V_H_11 CLL in *IgH.TE*μ mice. **(A)** Volcano plot showing *P*-values and fold changes in gene expression levels comparing V_H_11 and non-V_H_11 CLL from *IgH.TE*μ mice. Genes upregulated in non-V_H_11 CLL are indicated in red; genes upregulated in V_H_11 CLL in blue. Only significantly different genes (Benjamini-Hochberg adjusted *p*-value < 0.05) are shown. Genes indicated represent a subset of the validation gene set analyzed in a larger CLL (see Figure 7). **(B)** Oncogenic hallmark signatures enriched among differentially expressed genes in V_H_11 (*n* = 3) vs. non-V_H_11 (*n* = 3) CLL from *IgH.TE*μ mice.

When we performed differential gene expression analysis (focusing only on genes passing a stringent statistical filter of Benjamini-Hochberg false discovery rate corrected *P* < 0.05), we found 148 differentially expressed genes (Figure 5A; Supplementary Table 2). Of these genes, 59 genes were upregulated in V_H_11 CLL and 89 genes were upregulated in non-V_H_11 CLL. To identify biological processes that underlie the transcriptional differences between V_H_11 and non-V_H_11 CLL, we performed pathway enrichment analysis using the Molecular Signatures Database (MSigDB) (37). Genes upregulated in V_H_11 CLL were functionally enriched for an interferon-mediated response, active Wnt signaling and constitutively active RAF1 signaling (Figure 5B, Supplementary Table 3A). On the other hand, genes downregulated in V_H_11 CLL were involved in quite diverse pathways, including interleukin-, epidermal growth factor receptor (EGFR)-, vascular endothelial growth factor (VEGF)-mediated signaling, metabolic processes, hypoxia and the UV radiation-induced stress response (Figure 5B, Supplementary Table 3A).

Taken together, these data suggest that in addition to a different origin, V_H_11 and non-V_H_11 CLL subsets display distinct transcriptional signatures, signifying differential activity of key signaling pathways.

### Strong BCR dependence of V_H_11/Vκ14 CLL in *IgH.TEμ* mice

Next, we performed a PCA of the 148 differentially expressed genes between V_H_11 and non-V_H_11 CLL. To investigate the impact of T-cell-independent BCR stimulation and T-cell-dependent CD40 stimulation on differential gene expression, we included RNA-Seq gene expression values of the 148 genes from the unstimulated and αIgM-stimulated B cells described above, as well as previously reported gene expression values from anti-CD40/IL-4 stimulated follicular B-cells (α-CD40/IL4-B) (33).

**Figure 6:**
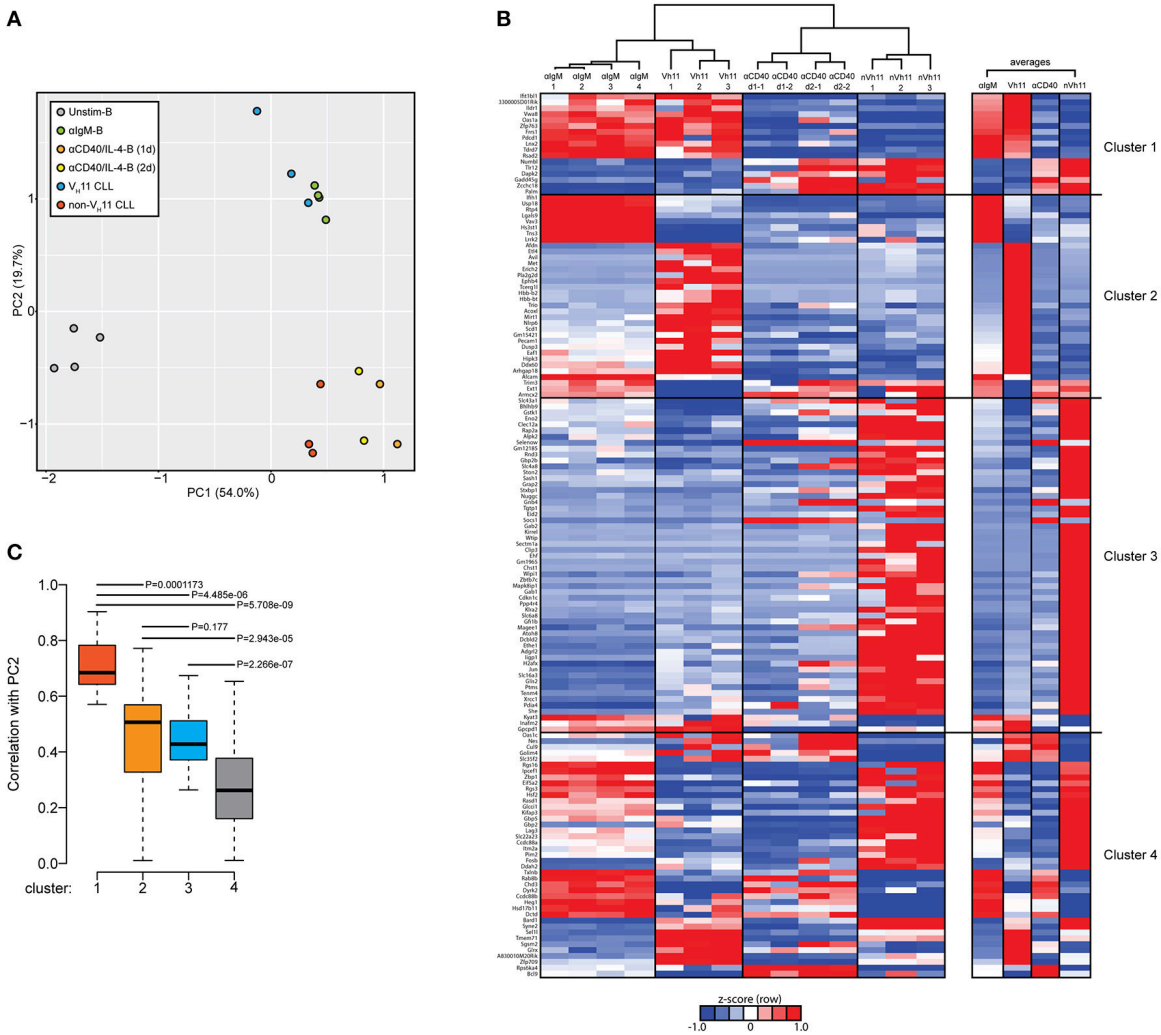
Genes discriminating V_H_11 from non-V_H_11 CLL show similarly distinct expression profiles in BCR or CD40-stimulated B-cells. **(A)** Principle component analysis (PCA) using the 148 differentially expressed genes defined in Figure 5A in unstimulated (*n* = 4, *black*), anti-IgM-stimulated (*n* = 4, *green*) WT splenic B cells, 1 day (*n* = 2, *orange*) or 2 day (*n* = 2, *yellow*) anti-CD40 plus IL-4 stimulated follicular B-cells (obtained from GSE77744), V_H_11-2^+^/V_k_14-126^+^ BCR (*n* = 3, *blue*) and non-V_H_11 (*n* = 3, *red*) BCR-expressing CLL from *IgH.TE*μ mice. **(B)** Hierarchical clustering analysis (top) and accompanying heat map showing differences in expression levels (RPKM, shown as row Z-scores) of the 148 gene signature in anti-IgM-stimulated (*n* = 4) WT splenic B cells, 1 day (*n* = 2) or 2 day (*n* = 2) anti-CD40 plus IL-4 stimulated follicular B-cells, V_H_11 and non-V_H_11 CLL from *IgH.TE*μ mice. Heatmap shown on the right shows average expression levels for each group. **(C)** Boxplot showing average correlation values of each of the four gene clusters shown in B with principal component 2 (PC2) from the PCA shown in A. *P*-values were calculated using a Mann-Whitney *U*-test.

The first principal component (PC1) separated both CLL groups and the two stimulated B cell subsets from unstimulated B cells, suggesting *IgH.TE*μ CLL cells share a transcriptional signature related to activated B-cell phenotypes. Interestingly, PC2 revealed a strong similarity between αIgM-stimulated B cells and V_H_11 CLL on one hand and between α-CD40/IL4-stimulated B cells and non-V_H_11 CLL on the other hand (Figure 6A). These findings indicate more prominent BCR stimulation in V_H_11 than in non-V_H_11 CLL B cells *in vivo* and are consistent with a dependence on T-cell help for non-V_H_11 CLL.

To identify the gene signature underlying the clustering of αIgM-stimulated B cells and V_H_11 CLL, as well as α-CD40/IL-4-stimulated B cells and non-V_H_11 CLL, we performed hierarchical clustering analyses to separate the 148 genes into 4 clusters (Figure 6B, Supplementary Table 3B). Cluster 1 consists of 17 genes that were highly correlated between αIgM-stimulated B cells and V_H_11 CLL and between α-CD40/IL-4-stimulated B cells and non-V_H_11 CLL. Pathway enrichment analysis (Supplementary Table 3C) on this cluster revealed overrepresentation of genes involved in interferon response and KRAS signaling. Clusters 2 (35 genes) and Cluster 3 (56 genes) consist of genes that were highly correlated only between α-CD40/IL-4-stimulated B cells and non-V_H_11 CLL or only between αIgM-stimulated B cells and V_H_11 CLL, respectively. These clusters were enriched for interferon response/PI3K-AKT signaling genes (cluster 2) or UV response, epithelial-mesenchymal transition, glycolysis, hypoxia, unfolded protein response genes (cluster 3) (Supplementary Table 3C). Finally, cluster 4 (enriched for genes involved in the reactive oxygen species pathway) represents genes with low or anti-correlated expression values between the stimulated B cells and CLL. Thus, genes from clusters 1 and 3 signify the clustering of αIgM-stimulated B cells and V_H_11 CLL, while genes from clusters 1 and 2 drive the clustering of α-CD40/IL-4-stimulated B cells and non-V_H_11 CLL (Figure 6B). This analysis was further validated by computing the average correlation strength for each of the four gene clusters with PC2 from our PCA (Figure 6B). Indeed, clusters 1 to 3 underlying the αIgM-B cells and V_H_11 CLL and the α-CD40/IL-4-B cells and non-V_H_11 CLL segregation—and particularly cluster 1 genes—showed significantly stronger correlation values with PC2 than cluster 4 (Figure 6C).

### Validation of V_H_11/non-V_H_11 CLL gene expression differences in mouse and human CLL

To further strengthen the existence of a unique transcriptional signature that differentiates V_H_11 and non-V_H_11 CLL B cells, we selected 24 robustly differentially expressed genes for validation. Some of these genes have already been shown to play a role in hematologic malignancies, including CLL (*Pim-2, Met, Rgs16, Ccdc88a, Zcchc18, Clip3*) (38–42), diffuse large B cell lymphoma, follicular lymphoma (*Vav3*) (43), acute lymphoblastic leukemia (ALL) (*Itm2a, Chst1*) (44, 45) or acute myeloid leukemia (AML) (*Chd3*) (46).

**Figure 7:**
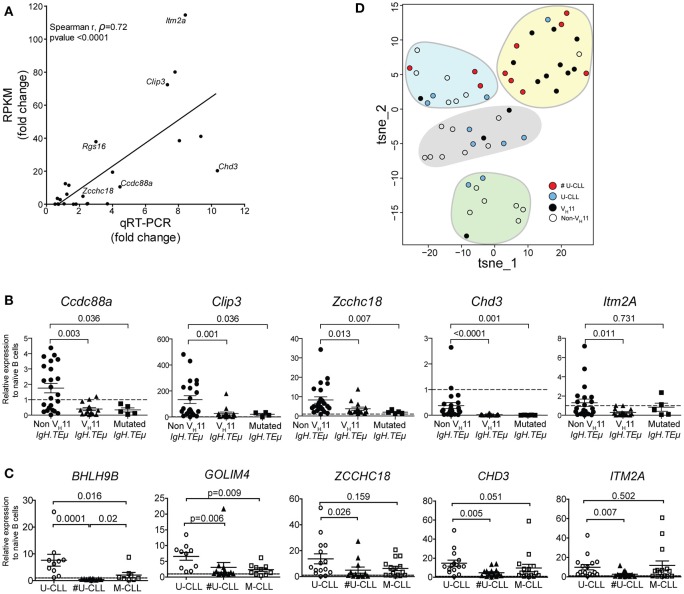
qRT-PCR validation of a subset of differentially expressed genes. **(A)** Correlation plot comparing fold-changes in expression between V_H_11 and non-V_H_11 CLL for 24 genes measured by RNA-Seq (RPKM) or qRT-PCR (spearman *r*, ρ = 0.72; *p* < 0.0001). The differentially expressed genes selected for further study are indicated. Characteristics of samples tested in qRT-PCR are provided in the Methods section **(B)** Expression of indicated genes as measured by qRT-PCR in V_H_11 (*n* = 15), non-V_H_11 (*n* = 23) and mutated (*n* = 5) CLL from *IgH.TE*μ mice. **(C)** Expression of indicated genes as measured by qRT-PCR in CLL cells from non-stereotypic U-CLL (*n* = 15), stereotypic U-CLL (#U-CLL, *n* = 14) and M-CLL (*n* = 15) patients. Bars in **(B,C)** represent mean ± SEM values. The expression values were calculated relative to expression in **(B)** naïve splenic WT B cells from mice (*n* = 4) or **(C)** naïve circulating B cells from healthy controls (*n* = 3), both of which were set to 1 (*dashed line*). Numbers indicate *p*-values (Mann-Whitney *U*-test). **(D)** t-SNE clustering analysis of the expression values for 13 signature genes (from Supplementary Table 7) using dCT values obtained by qRT-PCR for non-sterotypic (#U-CLL, *n* = 10) and stereotypic (U-CLL, *n* = 10) human U-CLL and non-V_H_11 (*n* = 21) and V_H_11 (*n* = 14) CLL from *IgH.TE*μ mice, as indicated. Expression values were converted to Z-scores separately for mouse and human datasets to allow combined t-SNE analysis.

Expression levels were validated by quantitative real-time PCR (qRT-PCR) in an extended cohort of 15 V_H_11 and 23 non-V_H_11 primary CLL from *IgH.TE*μ mice. Naïve Splenic B cells from wild type mice (*n* = 4) were included as controls. Comparison of RNA-Seq (RPKM) and qRT-PCR expression fold changes between the two CLL groups revealed highly correlated trends for these 24 genes (spearman correlation *r* = 0.72; *p* < 0.0001), validating our RNA-Seq analysis when extrapolated to a larger *IgH.TE*μ CLL cohort (Figure 7A). qRT-PCR validation showed that 11/24 (~46%) of the selected genes were significantly different (*p* < 0.05) between V_H_11 and non-V_H_11 CLL (Supplementary Table 4, Figure 7B). Additionally, 7/24 (~29%) genes were significantly different (*p* < 0.05) between non-V_H_11 CLL from *IgH.TE*μ and *IgH.TE*μ*.Siglec-G*^−/−^ mice, which might be related to the altered V_H_ usage in *Siglec-G*^−/−^ mice or the early disease onset in *IgH.TE*μ*.Siglec-G*^−/−^ mice.

Expression of five of these 13 genes that were significantly upregulated in non-V_H_11 CLL vs. V_H_11 CLL (C*cdc88a, Clip3, Zcchc18, Chd3, Itm2a*) was also evaluated in five mutated *IgH.TE*μ CLL, defined by < 97% IghV germline identity [Supplementary Table 1 and ter Brugge et al. (22)]. Interestingly, qRT-PCR analysis showed that four out of five tested genes (except *Itm2a*) were expressed at low levels in mutated CLL, similar to V_H_11 CLL (Figure 7B). Thus, non-V_H_11 unmutated CLL in *IgH.TE*μ mice represent a unique subset that can be distinguished from V_H_11 unmutated and from mutated CLL by a specific transcriptional signature. Furthermore, correlation analyses indicated that within the non-stereotypic subgroup in particular V_H_1 CLL represents the most heterogeneous CLL subgroup in *IgH.TE*μ mice (*n* = 16; average spearman *r*, ρ = 0.280; Supplementary Figure 3). In these analyses we also found that expression of these five genes is positively correlated in V_H_11 CLL (*n* = 15; average spearman *r*, ρ = 0.537) and in the small non-V_H_11/non-V_H_1 CLL subgroups (*n* = 6; average spearman *r*, ρ = 0.703) (Supplementary Figure 3).

Next, we evaluated the expression of the 13 signature genes in a panel of 44 human CLL samples (15 non-stereotypic U-CLL, 14 stereotypic U-CLL, 15 M-CLL, Supplementary Table 5) by qRT-PCR. Hereby, 6/13 (~46%) genes (*CCDC88A, CLIP3, ZCCHC18, CHD3, ITM2A, GOLIM4*) were significantly higher expressed in all three CLL subsets than in naïve B-cells from healthy individuals, suggesting a role for these genes in CLL (Figure 7C, Supplementary Table 6). Expression of CLIP3 was significantly higher in non-stereotypic than M-CLL. Expression of ZCCHC18, CHD3, GOLIM4, BHLH9B, and ITM2A was significantly higher in non-stereotypic U-CLL compared to stereotypic U-CLL (Figure 7C).

To compute any parallel between stereotypic and heterogeneous U-CLL from patients and *IgH.TE*μ mice, we performed t-SNE clustering analysis on expression values for the 13 signature genes (Figure 7D, Supplementary Table 6). We used dCT values obtained by qRT-PCR for non-sterotypic (#U-CLL, *n* = 10) and stereotypic (U-CLL, *n* = 10) U-CLL as well as for non-V_H_11 (*n* = 21) and V_H_11 (*n* = 14) CLL from *IgH.TE*μ mice. Interestingly, 7/10 stereotypic U-CLL clustered with 10/14 V_H_11 CLL (Figure 7D). Conversely, non-stereotypic human U-CLL and mouse non-V_H_11 CLL showed a more heterogeneous distribution into several clusters largely devoid of stereotypic human U-CLL or mouse V_H_11 CLL.

Taken together, we conclude that differences in the expression of these signature genes in heterogeneous U-CLL, stereotyped U-CLL and M-CLL were partly overlapping between human CLL and the corresponding CLL subgroups in our *IgH.TE*μ *CLL* mouse model.

## Discussion

In this report, we investigated the role of antigenic pressure and BCR signaling thresholds on clonal selection of CLL cells in the *IgH.TE*μ CLL mouse model. We found that U-CLL tumors that develop in these mice can be classified into two different groups based on their *IghV* usage. The stereotypic V_H_11-2/V κ14-126 CLL subset recognized the PtC self-antigen, developed independently of T cell help or GC formation and represented a somewhat more aggressive type of CLL. Proportions of V_H_11/Vκ14-expressing CLL were increased in the absence of functional germinal centers in *IgH.TE*μ mice deficient for CD40L or activation-induced cytidine deaminase. Conversely, *in vivo* T cell-dependent immunization decreased the proportions of V_H_11/Vκ14-expressing CLL. Mice were immunized at 10–12 weeks of age, with a secondary immunization at 15–17 weeks of age. In a proportion of mice at these time points, CLL cells become detectable in peripheral blood (Figure 1). In our immunization model the onset or frequency of CLL was not altered, but we cannot exclude that there will be effects on CLL onset or disease progression when immunizations are performed at a different age.

Consistent with the observed effects of defective germinal center function or robust T-cell dependent immunization on V_H_ usage in CLL, PCA of a gene signature comprised of 148 genes differentially expressed between V_H_11 and non-V_H_11 CLL revealed that V_H_11 and non-V_H_11 CLL clustered with BCR-stimulated and anti-CD40-stimulated B cells, respectively.

The unmutated V_H_11 CLL cells parallel B-1 cells, because these also have a restricted BCR repertoire, may recognize auto-antigens including PtC, and produce natural IgM antibodies in the absence of T cell co-stimulation (12). In concordance, it was recently shown that peritoneal CD5^+^ B-1 cells generated early during fetal or neonatal development, increase in number over time and can progress into CLL in aged mice (47, 48). Interestingly, CLL development in these mice was linked to the expression of a restricted BCR repertoire (V_H_Q52/V κ9 or V_H_3609/V κ21, reactive toward non-muscle myosin-IIA or Thy-1, respectively) independent of CD40 signaling. Hereby, expression of the *E*μ*-TCL1* transgene enhanced aggressiveness of the disease.

Non-V_H_11 CLL, on the other hand, consisted of tumors with heterogeneous *IghV/IglV* expression and CDR3 length, lacking affinity for PtC. Although these tumors were T-cell dependent, strongly reduced in the absence of functional GCs, their BCRs were not hypermutated (<3%). This is in line with findings in human U-CLL, indicating that U-CLL cells can recognize both TD and TI autoantigens that have relocated to the external cell surface during apoptosis (11, 13, 14). Our observations are also consistent with gene expression profiling studies suggesting that U-CLL reflect memory B cells (49). In contrast, more recent transcriptome analyses revealed that U-CLL resemble mature pre-GC CD5^+^CD27^−^ B cells, while M-CLL resembles a distinct, previously unrecognized, CD5^+^CD27^+^ post–GC B cell subset (18). Our findings imply that in mice unmutated CLL can be derived from (i) T cell-independent B-1 cells (e.g., PtC-recognizing V_H_11-2/Vκ14-126) or (ii) from B cells that recognize their antigen in the presence of cognate T-cell help and are activated without SHM. This latter group of T cell-dependent unmutated CLL displayed an expression signature, as defined by 13 genes including the *CCDC88A-CLIP3-ZCCHC18-CHD3-ITM2A* module, that is not only different from TI unmutated CLL, but also from mutated CLL in the *IgH.TE*μ mouse model. Moreover, we found evidence that this expression signature may be partly associated with non-stereotypic human U-CLL, suggesting that the development of human U-CLL can also be TD. Such TD U-CLL may derive from B cells involved in an extra-follicular response or alternatively may be related to auto-antibody producing B cells in mice that were shown to recognize TD antigens, mount a rapid IgM response and enter GCs, but do not develop into IgG-expressing plasma cells (50, 51). Although our data suggest a role for T-cell help in human non-stereotypic U-CLL pathophysiology, further investigation is required to translate our findings to human disease. Such studies should include expression profiling of (1) large CLL patient cohorts containing a wide range of stereotypic and non-stereotypic U-CLL samples and (2) activated B cells that received various stimulations including anti-CD40.

Gene expression profiling revealed a set of genes that distinguish V_H_11 from non-V_H_11 CLL and are similarly regulated in BCR or CD40-stimulated cells, respectively. This observation probably reflects differences in supporting external cues: pathways induced by interleukin or growth factor-mediated signaling were specifically upregulated in non-V_H_11 CLL. These include the regulator of G-protein signaling 16, *Rgs16*, which is upregulated in autoimmune B cells of BXD2 mice and enhances GC formation by the canonical NF-κB pathway, signifying the post-GC origin of non-V_H_11 CLL (52, 53). Second, the actin-binding protein *Ccdc88a*, which plays a role in cytoskeletal remodeling and cell migration following activation of Akt downstream of EGFR (54) and can also enhance Akt signaling (42, 55). Third, integral membrane protein 2A (*Itm2a*) is a type II integral membrane protein that has been associated with an enhanced GATA3-mediated regulatory network in B ALL (56). *Chd3* encodes a chromatin remodeler with unexplored function in lymphocytes.

On the other hand, Wnt-associated genes were specifically upregulated in V_H_11 tumors, which is interesting because the BTK-inhibitor ibrutinib restrains Wnt signaling in CLL (57). Although the function of several other upregulated genes is currently unknown, *Zcchc18* has been associated with a CLL-specific transcriptomic signature (42) and *Clip3* was differentially regulated in a CLL patient undergoing spontaneous regression (58). Notably, many gene sets or pathways were active in both CLL subsets, including high expression levels of MET receptor tyrosine kinase, which prolongs CLL cell survival through STAT3 and AKT phosphorylation (40, 59). This could contribute to the enhanced constitutive activation of the p-Akt/p-S6 pathway in *IgH.TE*μ CLL as reported previously (23, 24). Additionally, genes involved in KRAS signaling were highly expressed in both CLL subsets, consistent with its essential role in B cell lymphopoesis (60), particularly for B-1 cells recognizing PtC (61).

Our data also indicated that availability of T cell help and GC formation did not affect tumor incidence or onset. In contrast, the finding of a significantly earlier CLL incidence of mainly the non-V_H_11 type in *IgH.TE*μ*.Siglec-G*^−/−^ and *IgH.TE*μ*.E-Btk-2* mice suggests that BCR signaling thresholds are a key factor in determining CLL disease course. Yet, the appearance of V_H_11 CLL in these mouse lines may indicate a substantial selective advantage of these clones, because in *Siglec-G*^−/−^ and *E-Btk-2* transgenic mice the frequency of PtC-recognizing cells within the B-1 cell population is very low (28, 36).

In conclusion, we found that the formation of a major subset of unmutated CLL in *IgH.TE*μ mice is dependent on T cell signals. Our findings therefore provide a mechanistic explanation for the role of B-cell intrinsic factors, in particular BCR signaling, as well as extrinsic factors such as T cell help and support from the tumor microenvironment, in shaping the repertoire of CLL in mice. These findings are of potential clinical relevance, because B-cell extrinsic signals may reflect effective targets for novel therapeutic strategies in CLL patients.

## Author contributions

SPS designed the research studies, performed experiments, analyzed the data, and wrote the manuscript. MdB, SP, RM, and MdA performed experiments and analyzed the data. RS analyzed RNA sequencing data and contributed to writing the manuscript. AL and LN contributed to the research design and the writing of the manuscript. RH contributed to the research design and the writing of the manuscript and supervised the study. All co-authors approved the final manuscript.

### Conflict of interest statement

SP was affiliated to Erasmus MC Rotterdam whilst this study was completed. After finishing the work, he founded the company EpiExpressions. The remaining authors declare that the research was conducted in the absence of any commercial or financial relationships that could be construed as a potential conflict of interest.
