# Supplementary material for: Identification of Distinct Unmutated Chronic Lymphocytic Leukemia Subsets in Mice Based on Their T Cell Dependency
Source: Front Immunol. 2018 Sep 13;9:1996. doi: 10.3389/fimmu.2018.01996 (PMC6146083; doi:10.3389/fimmu.2018.01996)
Supplement: Supplementary file 1 [file Table_1.DOC]

Suppl. Table 1 : B-cell receptor characteristics of CLLs from IgH.TEµ mice.


Tumor code		IgH		HCDR3	Length	% BCR
germline
identity	IgL		LCDR3	Length	
	VH	DH	JH				VL	JL			
IgH.TEµ											
E-09	1-5	4-1	3	CTRMELSWFAYW	12	100%	6-17	5	CQQHYSTPPTF	11	
E-14	1-5	4-1	3	CTRMELSWFAYW	12	100%	1-135	1	CWQGTHFPQWTF	12	
E-19	1-9	1-1	4	CARRWFQYAMDYW	13	96.97%	14-126	4	CLQHGESPFTF	11	
C-ECD4-03	1-9	2-4	1	CARHYDQGWYFDVW	14	99.60%	4-91	5	CQQGSSIPRTF	11	
ESG-C07	1-9	1-1	2	CAREEKFGITTVVATKHYFDYW	22	93.51%	17-121	1	CLQSDNLPRTF	11	
EX-04	1-15	2-5	2	CTRYYRNYCGLL*	13	98.71%	9-124	2	CLQHASSPPTF	11	
C87	1-26	4-1	4	CATGYYAMDYW	11	100%	5-39	5	CQNGHSFPLTF	11	
E-22	1-39	2-4	2	CAGTYYDYDEWYFDYW	16	100%	6-15	4	n/a	3	
C89	1-47	n/a	3	CARGGFAYW	9	100%	2-109	1	CAQNLELPPF	10	
E-23	1-52	1-1	3	CARFYYGSSAWFAYW	15	99.63%	5-48	5	CQQSNSWPLTF	11	
E-24	1-52	2-1	1	CAIYYGNYWYFDVW	14	100%	1-117	1	CFQGSHVPWTF	11	
E-29	1-53	2-4	2	CARDYDYDYW	10	100%	6-15	2	CQQYNSYPYTF	11	
C-ECD4-05	1-53	n/a	1	CAREWYFDVW	10	100%	5-39	1	CQNGHSFPPTF	11	
C92	1-55	2-3	4	CARGNDAHCMDYW	13	100%	6-17	4	CQQHYSTPPTF	11	
E-28	1-62-2	1-1	2	CARHEDYYGSYSFDYW	16	100%	2	2	CALWYSTHYVF	11	
ESG-C15	2-2	1-2	4	CARLSHYYGYDYAMDYW	17	99.12%	14-126	4	CLQHGESPFTF	11	
E-27	3-6	1-1	2	CAREDFHYYYGSYYFDYW	18	100%	2-137	2	CMQHLEYPYTF	11	
C77	3-6	2-5	1	CANSNYVSYWYFDVW	15	100%	5-39	5	CQNGHSFPLTF	11	
E-25	5-6	1-1	2	CARHYYGSGYYFDYW	15	100%	5-39	5	CQKGNSSPLTF	11	
ESG-C06	9-3-1	1-1	2	CASLYGYFDYW	11	91.77%	4-63	2	CFQGSGYPLTF	11	
E-15	9-3	3-1	4	CARYSNYYAMDYW	13	100%	4-59	2	CQQWSSNP#YTF	12	
E-08	14-2	2-3	2	CAPDGYYFDYW	11	100%	6-15	5	CQQYNSYPLTF	11	
C-ECD4-02	14-4	1-1	3	CTTDYYGSGYFAYW	14	100%	1-135	1	CWQGTHFPQWTF	12	
E-32	11-2	1-1	1	CMRYGGYWYFDVW	13	100%	14-126	2	CLQHGESPYTF	11	
E-07	11-2	2-1	1	CMRYGNYWYFDVW	13	100%	14-126	4	CLQHGESPFTF	11	
E-20	11-2	2-1	1	CMRYGNYWYFDVW	13	99.21%	14-126	2	CLQHGESPYTF	11	
EX-02	11-2	2-1	1	CMRYGNYWYFDVW	13	100%	14-126	2	CLQHGESPYTF	11	
EX-03	11-2	2-1	1	CMRYGNYWYFDVW	13	100%	14-126	4	CLQHGESPFTF	11	
C-E41K-02	11-2	2-1	2	CMRYDNYYYFDYW	13	100%	14-126	2	CLQHGESPYTF	11	
ESG-C03	11-2	2-1	1	CMRYGNYWYFDVW	13	100%	14-126	4	CLHYCQRPFTF	11	
ESG-C05	11-2	2-1	1	CMRYGNYWYFDDW	13	99.51%	14-126	4	CLQHGESPFTF	11	
ESG-C11	11-2	2-1	1	CMRYGNYWYFDAW	13	100%	14-126	4	CLQHGESPFTF	11	
ESG-C13	11-2	2-1	1	CMRYGNYWYFDVW	13	100%	14-126	2	CLQHGESPYTF	11	
E-01	11-2	2-5	1	CMRYSNYWYFDVW	13	98.88%	14-126	2	CLQHGESPYTF	11	
E-06	11-2	2-5	1	CMRYSNYWYFDVW	13	100%	14-126	2	CLQHGESPYTF	11	
C-ECD4-01	11-2	2-5	1	CMRYSNYWYFDVW	13	100%	14-126	2	CLQHGESPYTF	11	

C-ECD4-04	11-2	2-5	1	CMRYSNYWYFDVW	13	100%	14-126	4	CLQHGESPFTF	11	
C-84	11-2	2-5	1	CMRYSNYWYFDVW	13	100%	14-126	2	CLQHGESPYTF	11	
ESG-C08	n/a	n/a	n/a	n/a	3	n/a	14-126	4	CLQLVESPFTF	11	
ESG-C12	n/a	n/a	n/a	n/a	3	n/a	14-126	4	CLQHGESPFTF	11	
ESG-C16	n/a	n/a	n/a	n/a	3	n/a	14-126	1	CLQHGESPWTF	11	
IgH.TEµ.AICDA-/-	
EA-08	1-26	2-13	2	CARKDYYFDYYC	12	99.09%	14-126	2	CLQHGESPYTF	11	
EA-10	1-50	4-1	2	CASPQLERDYW	11	99.64%	14-126	4	CLQHGESPFTF	11	
EA-07	1-78	1-1	2	CARYGPYYFDYW	12	99.21%	1-135	1	CWQGTHFPRWTF	12	
EA-09	2-2	2-3	4	CARNPDGYYVSYYAMDYW	18	99.63%	n/a	n/a	na	2	
EA-01	11-2	1-1	1	CMRYGSYWYFDVW	13	100%	14-126	4	CLQHGESPFTF	11	
EA-11	11-2	2-1	1	CMRYGNYWYFDVW	13	100%	14-126	4	CLQHGESPFTF	11	
EA-12	11-2	2-1	1	CMRYGNYWYFDVW	13	100%	14-126	4	CLQHGESPFTF	11	
EA-05	11-2	2-3	2	CMRYDGYYYYFDYW	14	99.25%	14-126	2	CLQHGESPYTF	11	
EA-06	11-2	2-3	1	CMRYDDGYWYFDVW	14	100%	14-126	1	CLQHGESPWTF	11	
EA-02	11-2	2-5	1	CMRYSNYWYFDVW	13	100%	14-126	2	CLQHGESPYTF	11	
EA-03	11-2	2-5	1	CMRYSNYWYFDVW	13	100%	14-126	1	CLQHGESPWTF	11	
EA-04	11-2	2-5	1	CMRYSNYWYFDVW	13	99.63%	14-126	2	CLQHGESPYTF	11	
EA-07	11-2	2-5	1	CMRYSNYWYFDVW	13	99.59%	1-135	1		0	
IgH.TEµ.CD40-/-	
ECD40-01	11-2	2-1	1	CMRYGNYWYFDVW	13	100%	14-126	2	CLQHGESPYTF	11	
ECD40-02	11-2	2-1	1	CMRYGNYWYFDDW	13	100%	14-126	4	CLQHGESPFTF	11	
ECD40-03	11-2	2-5	1	CMRYSNYWYFDVW	13	100%	14-126	2	CLQHGESPYTF	11	
ECD40-04	11-2	2-1	1	CMRYGNYWYFDVW	13	100%	14-126	4	CLQHGESPFTF	11	
ECD40-05	11-2	2-5	2	CMRYSNYWYFDYW	13	100%	14-126	2	CLQHGESPYTF	11	
ECD40-06	11-2	2-5	1	CMRYSNYWYFDVW	13	100%	14-126	2	CLQHGESPYTF	11	
ECD40-07	11-2	2-1	1	CMRYGNYWYFDVW	13	100%	14-126	2	CLQHGESPYTF	11	
IgH.TEµ.TD	
ED-07	1-7	1-1	2	CGRYYYGSSYYFDYW	15	100%	9-120	2	CLQYASSPYTF	11	
ED-09	1-9	2-3	4	CARDDGTTWSMDYW	14	100%	n/a	n/a	n/a	3	
ED-03	1-19	n/a	3	CATAYW	6	100%	4-59	1	CQQWSSNPQWTF	12	
ED-01	1-39	2-4	1	CAGVYYDYDEWYFDVW	16	100%	8-30	2	n/a	3	
ED-02	1-39	2-4	1	CARIYYDYDDWYFDVW	16	100%	8-30	2	CQQYYSYPYTF	11	
ED-06	1-55	1-1	4	CARRDYYGSSYAMDYW	16	99.27%	n/a	n/a	n/a	3	
ED-04	2-2	1-1	4	CARKTTVVDYYDMDYW	16	100%	14-126	4	CLQHGESPFTF	11	
ED-10	7-3	2-4	2	CARYDYDYW	9	100%	6-15	5	CQQYNSYPLTF	11	
ED-05	9-03	2-1	2-1	CARFLSYGYLEYW	13	100%	4-68	5	CQQWSSNPLTF	11	
ED-08	11-2	2-1	1	CMRYGNYWYFDVW	13	100%	14-126	4	CLQHGESPFTF	11	
EI-07	11-2	2-1	1	CMRYGNYWYFDVW	13	100%	14-126	4	CLQHGESPFTF	11	
IgH.TEµ.E-BTK-2	
E41K-03	1-26	2-2	2	CARGVTTRGFDYW	13	100%	4-59	2	CQQWSSNPYTF	11	
E41K-15	1-55	2-1	2	CASGNYYW	8	99.13%	n/a	n/a	n/a	3	
E41K-13	1-61	1-1	3	CARSIYYYGSSWFAYW	16	100%	6-17	5	CQQHYSTPLTF	11	
E41K-11	1-72	1-1	2	CARSRGGGYYYGSSPFDYW	19	99.54%	n/a	n/a		0	
E41K-10	1-81	2-5	1	CARGATYYSNYGWYFDVW	18	99.54%	1-135	5	CWQGTHFPLTF	11	
E41K-06	14-2	2-1	2	CARYGNYFDYW	11	93.07%	4-61	4	CQQYHSYPFTF	11	
E41K-17	14-2	2-1	2	CARYGNYFDYW	11	99.06%	5-39	2	CQNGHSFPYTF	11	
E41K-09	11-2	1-3	1	CMRYSSYWYFDVW	13	100%	14-126	4	CLQHGESPFTF	11	
IgH.TEµ.SiglecG-/-	
											
ESG-T01	1-62	1-1	2	CARRLYYYAMDYW	13	100%	3-10	2	CQQNNEDPYTF	11	
ESG-T02	1-62-2	3-2	4	CARHEGGKLRQLRPYAMDYW	20	100%	17-121	2	CLQSDNLPLTL	12	
ESG-T10	2-2	1-1	4	CARGGYRLYYYAMDYW	16	99.12%	14-126	4	CLQHGESPFTF	11	
ESG-T05	2-9	1-3	4	CYKCAGDYNYYGKFYC*	17	-	4-59	5	CQQWSSNPPTF	11	
ESG-T08	5-6	2-4	4	CARERVITTCGYYAMDYW	18	100%	1	1	CLQSDNLPLTF	11	
ESG-T14	5-6-4	1-1	1	CTRDRGPFITTVAAKGWYFDVW	22	100%	17-121	2	CLQSDNLPLTF	11	
ESG-T06	5-12-1	2-2	1	CARGRIYYGYDWYFDVW	17	99.57%	3-10	2	CQHNKEVPGTF	11	
ESG-T07	14-3	1-1	3	CASVYYYGSSYWFAYW	16	100%	19-93	1	CLQYDNLWTF	10	
ESG-T03	11-2	1-1	1	CMRYGSSYWYFDVW	14	100%	14-126	1	CLQHGESPWTF	11	
ESG-T12	11-2	2-1	1	CMRYGNYWYFDVW	13	100%	14-126	4	CLQHGESPFTF	11	
ESG-T11	n/a	n/a	n/a	n/a	3	n/a	2	2	CALWYSTHYVF	11	
