# Supplementary material for: Identification of Distinct Unmutated Chronic Lymphocytic Leukemia Subsets in Mice Based on Their T Cell Dependency
Source: Front Immunol. 2018 Sep 13;9:1996. doi: 10.3389/fimmu.2018.01996 (PMC6146083; doi:10.3389/fimmu.2018.01996)
Supplement: Supplementary file 2 [file Table_2.DOC]

Suppl. Table 2 : (A) Expression values (De seq) for genes up regulated in non-VH11 CLL (n=3) compared to VH11 CLL (n=3) from IgH.TEµ CLL mice.


Gene ID		VH11			Non-VH11		p-value	adj. p-value	
	E-06	EA-02	EA-04	ET-06	E-15	E-29			
Itm2a	4.88	4.92	5.01	7.79	8.75	8.12	7.26E-31	8.97E-27	
Clip3	5.03	4.85	5.09	7.48	8.36	8.03	1.55E-26	9.57E-23	
Armcx2	4.87	4.94	4.64	6.69	7.79	8.74	6.39E-15	1.97E-11	
Zcchc18	8.97	8.62	8.57	10.30	9.99	10.36	1.44E-11	1.98E-08	
Slc16a3	6.70	6.56	6.66	8.04	7.61	8.03	7.21E-10	8.09E-07	
Kifap3	4.83	4.63	4.85	5.81	6.91	7.53	1.48E-09	1.53E-06	
Gm1965	5.02	4.52	4.66	7.56	6.04	7.81	1.66E-09	1.58E-06	
Chd3	6.63	7.16	6.72	8.13	9.54	10.31	1.90E-09	1.68E-06	
Eif5a2	4.89	5.34	4.85	7.49	6.33	7.38	5.39E-09	4.16E-06	
Dcbld2	6.74	6.90	6.62	7.98	7.76	8.27	1.28E-08	8.81E-06	
Hsf2	5.50	5.60	5.97	6.93	7.50	7.17	3.07E-08	1.99E-05	
Tlr12	5.31	5.56	6.26	8.16	8.20	7.89	3.32E-08	2.05E-05	
Gm12185	4.94	4.81	4.89	5.73	5.81	5.84	5.65E-08	3.17E-05	
Hs3st1	5.40	4.88	5.04	7.09	6.36	6.72	5.58E-08	3.17E-05	
Rgs16	4.34	4.18	4.25	4.85	6.16	6.37	6.80E-08	3.65E-05	
Ccdc88a	6.31	6.51	7.10	7.96	9.19	8.76	7.93E-08	4.08E-05	
Stxbp1	6.86	7.07	6.94	7.88	9.48	8.70	2.27E-07	0.000107841	
Jun	11.70	11.56	11.13	12.41	13.27	13.13	4.10E-07	0.000181039	
Tns3	5.49	6.26	6.32	8.46	7.48	8.20	6.61E-07	0.000268405	
Tgtp1	4.12	3.99	4.08	5.11	4.77	5.40	6.74E-07	0.000268405	
Bhlhb9	6.49	5.29	5.12	8.40	9.27	9.34	6.60E-07	0.000268405	
Atoh8	5.41	5.19	5.29	5.91	7.15	7.27	7.55E-07	0.000291503	
Gbp2	10.68	10.37	10.34	11.46	11.35	11.36	8.14E-07	0.000304616	
H2afx	11.52	11.10	11.25	12.14	12.44	12.31	1.20E-06	0.000436441	
Slc4a8	7.28	7.66	7.49	8.48	8.99	8.45	1.62E-06	0.000570073	
Fosb	10.88	10.57	10.66	12.07	11.42	12.15	2.39E-06	0.00075843	
Trim3	7.06	7.23	6.79	8.09	7.96	8.10	2.65E-06	0.000798721	
Glis2	6.44	6.23	6.37	7.74	7.64	7.03	2.64E-06	0.000798721	
Dyrk2	4.41	4.51	4.33	6.57	7.32	4.77	2.75E-06	0.000808864	
Zbtb7c	4.40	4.44	4.38	4.68	7.00	6.75	2.82E-06	0.000810693	
Eno2	5.37	5.52	6.09	7.95	8.00	6.59	3.06E-06	0.000859968	
Lag3	6.58	6.18	6.99	7.86	8.02	8.45	3.21E-06	0.000881573	
Slc43a1	5.32	5.34	5.91	7.55	6.46	7.38	4.79E-06	0.001258279	
Mapk8ip1	4.74	4.71	5.07	6.10	6.57	5.70	4.77E-06	0.001258279	
Gnb4	4.34	4.51	4.72	5.40	7.10	6.65	5.53E-06	0.001393159	

Bard1	10.19	10.07	10.07	10.72	10.83	10.84	5.76E-06	0.001422014	
Gab2	5.56	6.12	6.21	6.79	8.06	8.02	6.14E-06	0.001486478	
Rap2a	5.70	6.13	6.57	7.55	7.88	7.64	7.81E-06	0.0017873	
Ethe1	8.40	8.27	8.98	9.69	10.15	9.72	8.94E-06	0.001987385	
Palm	7.05	6.97	7.39	8.18	8.17	7.99	1.04E-05	0.00221295	
Chst1	4.71	5.01	5.57	7.48	6.97	8.80	1.15E-05	0.002406911	
Rgs3	6.08	6.80	6.73	7.56	8.11	8.15	1.55E-05	0.003095186	
Wtip	4.09	4.11	4.29	4.42	6.65	6.73	1.84E-05	0.003498247	
Nuggc	3.55	3.51	3.56	4.12	4.00	4.42	2.52E-05	0.004573767	
Gstk1	4.55	4.24	4.27	5.08	5.26	6.31	3.05E-05	0.005464537	
Lrrk2	4.39	4.46	4.69	6.49	8.51	4.87	3.11E-05	0.005481953	
Ext1	4.70	5.43	5.23	6.17	7.92	7.92	4.43E-05	0.007390607	
Alpk2	6.20	6.36	6.22	6.87	7.02	7.25	6.47E-05	0.01038273	
Sectm1a	3.73	3.69	3.86	5.67	7.44	4.22	6.40E-05	0.01038273	
Vav3	4.31	4.30	4.53	4.60	6.09	6.48	6.76E-05	0.010468829	
Ipcef1	4.70	4.69	5.62	8.04	6.25	8.20	7.51E-05	0.011170042	
She	5.59	5.82	6.13	6.88	7.32	6.64	9.74E-05	0.013989469	
Zbp1	9.30	9.80	9.30	10.32	10.48	10.34	0.000101	0.014344109	
Gab1	4.03	4.11	4.17	4.38	6.49	5.18	0.000113	0.015652939	
Grap2	10.16	9.94	9.84	10.45	11.21	11.50	0.000128	0.016632321	
Magee1	6.72	6.48	6.39	7.21	7.34	7.84	0.000128	0.016632321	
Clec12a	5.07	5.34	5.85	6.89	6.94	6.62	0.000124	0.016632321	
Rnd3	3.68	3.73	3.72	4.25	4.99	4.05	0.000126	0.016632321	
Xrcc1	11.52	11.37	11.82	12.16	12.61	12.96	0.000148	0.018502352	
Adgrl2	9.32	8.97	7.46	10.80	10.95	10.44	0.000147	0.018502352	
Rasd1	5.62	5.10	5.83	5.74	7.27	8.87	0.000165	0.020392725	
Selenow	10.16	10.70	10.24	11.11	11.46	11.20	0.000248	0.028670004	
Eid2	4.17	3.99	4.23	4.20	6.65	5.81	0.000248	0.028670004	
Iigp1	7.31	6.31	7.16	9.38	8.57	7.57	0.000264	0.030151238	
Socs1	7.08	7.05	7.55	8.28	8.07	8.04	0.000276	0.031278948	
Cdkn1c	4.20	4.10	4.15	4.33	5.45	5.11	0.000281	0.031584949	
Ppp4r4	4.94	4.64	4.99	4.95	7.28	6.42	0.000284	0.031591382	
Tenm4	7.03	8.33	7.94	9.80	9.07	9.13	0.000289	0.031912588	
Dapk2	4.83	5.37	5.01	5.80	6.33	6.27	0.0003	0.032474417	
Slc6a8	5.40	5.17	5.57	5.45	7.34	7.31	0.00031	0.033023264	
Syne2	11.31	11.62	11.41	12.06	12.11	12.03	0.000343	0.03590087	
Gbp2b	3.80	3.81	3.94	4.52	4.31	4.62	0.000347	0.0359857	
Glcci1	10.55	9.99	10.40	11.02	11.13	11.58	0.000366	0.037100747	
Ston2	5.08	5.46	5.57	6.71	6.73	5.86	0.000406	0.03918737	
Kirrel	3.84	3.70	3.63	3.76	6.24	6.65	0.000401	0.03918737	
Pim2	10.19	10.86	10.84	11.28	11.73	12.47	0.000437	0.040891252	

Pdia4	13.18	13.29	13.36	13.94	13.72	14.12	0.000454	0.042152825	
Numbl	5.97	6.02	6.12	6.47	6.84	7.04	0.000486	0.044194009	
Klra2	4.13	4.12	4.37	5.65	4.74	4.89	0.00048	0.044194009	
Gadd45g	5.89	5.52	6.41	6.44	8.85	7.17	0.000511	0.04552599	
Ehf	4.14	4.70	4.49	4.81	5.77	7.80	0.000512	0.04552599	
Bcl9	8.88	9.19	9.29	9.73	10.43	9.87	0.000522	0.046084919	
Gbp5	9.24	8.85	9.03	9.80	9.65	9.70	0.000535	0.046830215	
Slc22a23	5.31	5.41	5.31	6.18	5.70	6.48	0.000541	0.047022792	
Ptms	10.43	10.77	10.04	11.16	11.31	11.75	0.000565	0.048438638	
Ddah2	8.75	8.10	8.46	9.39	9.87	9.09	0.000571	0.048620608	
Gfi1b	4.26	4.33	4.49	5.20	4.67	5.73	0.000575	0.048650393	
Sash1	6.67	6.02	6.60	6.23	7.97	9.93	0.000589	0.049457287	
Wipi1	4.09	4.18	4.28	5.08	4.61	4.82	0.000593	0.049528884	

Suppl. Table 2 : (B) Expression values (De seq) for genes down regulated in non-VH11 CLL (n=3) compared to VH11 CLL (n=3) from IgH.TEµ CLL mice.

Gene ID		VH11		Non-VH11		p-value	adj. p-value	
	E-06	EA-02	EA-04	ET-06	E-15	E-29			
Ephb4	8.30	8.99	9.23	6.15	5.79	5.65	1.34E-20	5.53E-17	
Arhgap18	7.24	6.90	6.81	5.42	5.41	5.32	2.37E-14	5.86E-11	
Dctd	6.72	6.22	7.66	4.61	4.46	4.46	3.51E-13	7.23E-10	
Golim4	8.57	7.79	8.80	6.26	6.07	6.54	3.23E-12	4.98E-09	
Mirt1	8.93	8.89	9.13	7.55	7.31	7.71	3.07E-12	4.98E-09	
Frrs1	7.30	7.10	7.13	5.83	5.71	6.03	2.17E-10	2.68E-07	
Oas1a	6.70	6.71	6.80	5.40	4.95	5.07	2.93E-09	2.41E-06	
Tcerg1l	8.57	5.85	6.91	4.30	4.16	4.25	6.85E-09	4.98E-06	
Afdn	8.91	9.25	8.36	7.61	7.46	7.41	2.23E-07	0.000107841	
Rps6ka4	9.35	8.73	9.00	7.51	7.96	7.46	2.77E-07	0.000126726	
Met	11.44	9.07	10.48	7.76	8.66	7.77	2.05E-06	0.000704356	
Slc35f2	5.64	6.76	7.29	4.77	5.09	5.09	2.15E-06	0.000719354	
Lgals9	9.66	9.72	9.71	8.60	7.69	8.38	2.38E-06	0.00075843	
Rsad2	6.69	6.98	7.09	6.05	5.82	6.01	5.11E-06	0.001315252	
Trio	10.23	11.16	10.65	9.51	8.72	9.34	6.99E-06	0.001659764	
Sgsm2	9.35	9.29	9.30	8.65	8.27	8.41	7.55E-06	0.001758856	
3300005D01Rik	5.58	6.31	6.57	4.94	4.70	5.07	9.11E-06	0.001987385	
Pecam1	10.68	11.31	10.14	9.22	9.31	9.59	9.17E-06	0.001987385	
Sel1l	13.29	13.03	13.10	12.39	12.42	12.50	1.27E-05	0.00262063	
Txlnb	4.34	4.18	4.37	3.70	3.70	3.65	1.41E-05	0.002861754	
Ddx60	7.63	7.86	7.11	5.86	5.33	6.26	1.62E-05	0.003133865	
Heg1	11.20	10.96	11.27	10.37	10.43	10.45	1.61E-05	0.003133865	
Lnx2	10.47	10.48	10.53	9.75	9.84	9.47	1.87E-05	0.003498247	
Vwa8	9.86	10.21	9.96	9.25	9.29	9.05	2.38E-05	0.00437842	
Eaf1	11.08	11.28	11.17	10.55	10.52	10.59	3.93E-05	0.006828959	
Glrx	7.56	8.08	8.06	7.11	6.74	6.94	4.18E-05	0.007175831	
Erich2	5.56	6.35	5.24	4.48	4.62	4.32	4.42E-05	0.007390607	
Acoxl	5.85	7.12	6.18	5.21	5.34	5.03	5.58E-05	0.009194496	
Hbb-bt	6.52	7.01	8.40	5.62	6.12	5.50	6.76E-05	0.010468829	
Hbb-b2	6.52	7.01	8.40	5.62	6.12	5.50	6.78E-05	0.010468829	
Rtp4	6.47	6.65	6.07	5.64	5.41	5.51	7.33E-05	0.011142398	
Ifih1	7.93	8.00	7.81	6.98	6.98	7.31	7.40E-05	0.011142398	
Tmem71	9.90	10.23	9.90	9.25	9.15	9.38	8.91E-05	0.013100572	
Ccdc88b	9.55	9.01	9.89	8.50	8.54	8.05	9.09E-05	0.013203545	
Usp18	7.57	7.51	7.29	6.81	6.57	6.71	0.000111	0.015543143	
Dusp3	8.59	10.11	8.16	7.41	7.76	7.19	0.000129	0.016632321	

Ifit1bl1	7.15	7.55	6.68	6.13	5.86	6.27	0.000128	0.016632321	
Rab8b	10.30	10.59	10.39	9.69	9.67	9.87	0.000128	0.016632321	
Inafm2	8.35	9.27	8.72	7.60	7.94	7.66	0.000137	0.017479161	
A830010M20Rik	9.70	10.00	9.42	8.45	8.98	8.82	0.000176	0.021537127	
Kyat3	6.88	5.86	7.52	5.01	5.69	5.05	0.000183	0.022126416	
Ildr1	5.02	7.29	6.03	4.88	4.84	4.71	0.00019	0.022782262	
Nes	4.19	5.83	6.33	4.18	4.10	4.21	0.000201	0.023826698	
Zfp763	6.79	6.36	6.43	5.80	5.78	5.81	0.000246	0.028670004	
Etl4	9.57	11.15	10.45	8.28	8.81	6.78	0.000293	0.031980144	
Gm15421	8.55	9.92	9.37	7.95	8.35	8.11	0.00031	0.033023264	
Cul9	5.26	8.61	6.88	5.52	5.32	5.11	0.000333	0.035139143	
Pla2g2d	4.46	6.13	6.17	4.44	4.45	4.25	0.000361	0.036946741	
Hipk3	12.33	12.12	11.40	10.87	10.70	11.20	0.000362	0.036946741	
Tdrd7	8.25	8.71	8.60	7.70	7.86	7.84	0.000373	0.037502256	
Avil	6.74	9.00	10.37	6.71	7.47	6.75	0.000397	0.03918737	
Hsd17b11	6.24	7.70	7.84	6.31	5.72	5.56	0.000405	0.03918737	
Scd1	15.07	14.48	13.70	13.02	13.33	13.49	0.000395	0.03918737	
Zfp709	9.15	8.79	8.82	8.23	8.14	8.33	0.000414	0.039648882	
Pdcd1	9.48	7.55	9.48	6.84	7.58	7.66	0.000435	0.040891252	
Alcam	10.36	10.44	10.21	9.81	9.74	9.52	0.000437	0.040891252	
Gpcpd1	12.45	12.36	12.32	11.86	11.93	11.85	0.000487	0.044194009	
Oas1c	7.48	7.12	7.47	6.66	6.23	6.64	0.000512	0.04552599	
Nlrp6	5.31	5.80	5.09	4.22	4.34	4.58	0.000564	0.048438638	
