# Supplementary material for: Identification of Distinct Unmutated Chronic Lymphocytic Leukemia Subsets in Mice Based on Their T Cell Dependency
Source: Front Immunol. 2018 Sep 13;9:1996. doi: 10.3389/fimmu.2018.01996 (PMC6146083; doi:10.3389/fimmu.2018.01996)
Supplement: Supplementary file 3 [file Table_3.DOC]

**Suppl. Table 3 (A):** List of enriched pathways (Oncogenic-Hallmark) for 148 differentially expressed genes in V_H_11 and non-V_H_11 CLL from *IgH.TEµ* mice

| **Gene Set Name** | **p-value** | **FDR q-value** | **Genes** |
| --- | --- | --- | --- |
| EGFR response | 4.41E-08 | 4.62E-06 | *Rgs16, Vav3, Gbp2, Tns3, Wipi1, Ext1, Ethe1* |
| Interferon response | 5.80E-08 | 4.62E-06 | *Rtp4, Rsad2, Ifih1, Usp18, Tdrd7, Ddx60* |
| Interleukin response | 1.15E-05 | 3.86E-04 | *Rgs16, Pim2, Rgs3, Socs1, Hs3st1, Ppp4r4,*  *Ehf, Fosb, Lag3, Cdkn1c, Socs1, She* |
| RAF upregulated | 1.30E-05 | 3.86E-04 | *Heg1, Rsad2, Glrx, Hsd17b11* |
| Hypoxia | 1.45E-05 | 3.86E-04 | *Eno2, Ext1, Cdkn1c, Jun, Hs3st1* |
| Glycolysis | 1.45E-05 | 3.86E-04 | *Eno2, Ext1, Chst1, Slc16a3* |
| UV response | 6.00E-05 | 1.43E-03 | *Eno2, Cdkn1c, Slc6a8, Fosb, H2afx* |
| WNT | 8.49E-05 | 1.84E-03 | *Usp18, Ifih1, Met* |
| VEGF | 1.65E-04 | 3.10E-03 | *Rgs3, Hs3st1, Eno2, Clip3, Itm2a* |

**Suppl. Table 3 (B) :** Genes belonging to individual cluster in Figure 6B.

| Cluster1 | Cluster2 | Cluster3 | Cluster4 |
| --- | --- | --- | --- |
| **Ifit1bl1** | **Ifih1** | **Bhlhb9** | **Nes** |
| **3300005D01Rik** | **Usp18** | **Gstk1** | **Cul9** |
| **Ildr1** | **Rtp4** | **Eno2** | **Golim4** |
| **Vwa8** | **Lgals9** | **Clec12a** | **Slc35f2** |
| **Oas1a** | **Vav3** | **Rap2a** | **Rgs16** |
| **Zfp763** | **Hs3st1** | **Alpk2** | **Ipcef1** |
| **Frrs1** | **Tns3** | **Selenow** | **Zbp1** |
| **Pdcd1** | **Lrrk2** | **Gm12185** | **Eif5a2** |
| **Lnx2** | **Afdn** | **Rnd3** | **Rgs3** |
| **Tdrd7** | **Etl4** | **Gbp2b** | **Hsf2** |
| **Rsad2** | **Avil** | **Slc4a8** | **Rasd1** |
| **Numbl** | **Met** | **Ston2** | **Glcci1** |
| **Tlr12** | **Erich2** | **Sash1** | **Kifap3** |
| **Dapk2** | **Pla2g2d** | **Grap2** | **Gbp5** |
| **Gadd45g** | **Ephb4** | **Stxbp1** | **Gbp2** |
| **Zcchc18** | **Tcerg1l** | **Nuggc** | **Lag3** |
| **Palm** | **Hbb-b2** | **Gnb4** | **Slc22a23** |
|  | **Hbb-bt** | **Tgtp1** | **Ccdc88a** |
|  | **Trio** | **Eid2** | **Itm2a** |
|  | **Acoxl** | **Socs1** | **Pim2** |
|  | **Mirt1** | **Gab2** | **Fosb** |
|  | **Nlrp6** | **Kirrel** | **Ddah2** |
|  | **Scd1** | **Wtip** | **Txlnb** |
|  | **Gm15421** | **Sectm1a** | **Rab8b** |
|  | **Pecam1** | **Clip3** | **Chd3** |
|  | **Dusp3** | **Ehf** | **Dyrk2** |
|  | **Eaf1** | **Gm1965** | **Ccdc88b** |
|  | **Hipk3** | **Chst1** | **Heg1** |
|  | **Ddx60** | **Wipi1** | **Hsd17b11** |
|  | **Arhgap18** | **Zbtb7c** | **Dctd** |
|  | **Alcam** | **Mapk8ip1** | **Bard1** |
|  | **Trim3** | **Gab1** | **Syne2** |
|  | **Ext1** | **Cdkn1c** | **Sel1l** |
|  | **Armcx2** | **Ppp4r4** | **Tmem71** |

| Cluster1 | Cluster2 | Cluster3 | Cluster4 |
| --- | --- | --- | --- |
|  | **Slc43a1** | **Klra2** | **Sgsm2** |
|  |  | **Slc6a8** | **Glrx** |
|  |  | **Gfi1b** | **A830010M20Rik** |
|  |  | **Magee1** | **Zfp709** |
|  |  | **Atoh8** | **Rps6ka4** |
|  |  | **Dcbld2** | **Bcl9** |
|  |  | **Ethe1** | **Sgsm2** |
|  |  | **Adgrl2** |  |
|  |  | **Iigp1** |  |
|  |  | **H2afx** |  |
|  |  | **Jun** |  |
|  |  | **Slc16a3** |  |
|  |  | **Glis2** |  |
|  |  | **Ptms** |  |
|  |  | **Tenm4** |  |
|  |  | **Xrcc1** |  |
|  |  | **Pdia4** |  |
|  |  | **She** |  |
|  |  | **Kyat3** |  |
|  |  | **Inafm2** |  |
|  |  | **Gpcpd1** |  |
|  |  | **Oas1c** |  |
|  |  |  |  |

**Suppl. Table 3 (C) :** List of enriched Hallmark pathways for genes from individual cluster in Figure 6B.

| **Gene Set Name** | **p-value** | **FDR q-value** | **Genes** |
| --- | --- | --- | --- |
| **Cluster 1** |  |  |  |
| Interferon alpha response | 2.87E-04 | 1.44E-02 | *Rsad2, Tdrd7* |
| Interferon gamma response | 1.21E-03 | 2.01E-02 | *Rsad2, Tdrd7* |
| Kras signaling | 1.21E-03 | 2.01E-02 | *Rsad2, Pdcd1* |
| **Cluster 2** |  |  |  |
| Interferon alpha response | 3.15E-07 | 7.53E-05 | *Rtp4, Ifih1, Usp18, Ddx60* |
| Interferon gamma response | 5.65E-06 | 6.75E-04 | *Rtp4, Ifih1, Usp18, Ddx60* |
| PI3K-AKT-mTOR Signaling | 1.75E-03 | 2.98E-02 | *Dusp3, Vav3* |
| **Cluster 3** |  |  |  |
| UV response | 1.36 E-05 | 6.8 E-04 | *Eno2, Cdkn1c, Slc6a8, H2afx* |
| Epithelial Mesenchymal transition | 3.42 E-05 | 8.56 E-04 | *Eno2, Jun, Slc6a8, Magee1* |
| Glycolysis | 8.23 E-04 | 8.23 E-03 | *Eno2, Chst1, Slc16a3* |
| Hypoxia | 8.23 E-04 | 8.23 E-03 | *Eno2, Cdkn1c, Jun* |
| IL-2 Stat5 Signaling | 8.23 E-04 | 8.23 E-03 | *Socs1, She, Cdkn1c* |
| IL-6 JAK Stat3 signaling | 2.9 E-03 | 2.42 E-02 | *Jun, Socs1* |
| Unfolded Protein Response | 4.84 E-03 | 3.46 E-02 | *H2afx, Wipi1* |
| **Cluster 4** |  |  |  |
| Reactive oxygen species | 7.64E-04 | 2.61E-02 | *Glrx, Ipcef1* |
|  |  |  |  |
