# Supplementary material for: Identification of Distinct Unmutated Chronic Lymphocytic Leukemia Subsets in Mice Based on Their T Cell Dependency
Source: Front Immunol. 2018 Sep 13;9:1996. doi: 10.3389/fimmu.2018.01996 (PMC6146083; doi:10.3389/fimmu.2018.01996)
Supplement: Supplementary file 4 [file Table_4.DOC]

**Suppl. Table 4:** Mean ± SEM expression of indicated genes in V_H_11 (n=15) or non- V_H_11 (n=23) tumors from IgH.TEµ mice and non-V_H_11 (n=8) tumors from IgH.TEµ,SiglecG^-/-^ mice. The values indicate relative expression (Mean ± SEM) to WT splenic B-cells.

|  | **Non-V_H_11**  ***IgH.TEµ***  **(n=23)** | **V_H_11**  ***IgH.TEµ***  **(n=15)** | **Non-V_H_11 *IgH.TEµ.SiglecG^-/-^***  **(n=8)** | **Kruskal-Wallis**  **Test** | **Dunn’s Multiple comparison**  **test between groups** |
| --- | --- | --- | --- | --- | --- |
|  | **(A)** | **(B)** | **(C)** |  |  |
| ***Chd3*** | 0.38 ± 0.12 | 0.02 ± 0.01 | 0.36 ± 0.07 | <0.0001 (***) | A vs B (***); B vs C (***) |
| ***Vav3*** | 0.28 ± 0.07 | 0.07 ± 0.04 | 0.07 ± 0.04 | 0.0009 (***) | A vs B (**) |
| ***Clip3*** | 133.2 ± 30.19 | 26.32 ± 11.62 | - 1. ± 4.05 | 0.0002 (***) | A vs B (**); A vs C (**) |
| ***Ccdc88a*** | 1.76 ± 0.30 | 0.39 ± 0.10 | 1.64 ± 0.17 | 0.0007 (***) | A vs B (**) B vs C (**) |
| ***Trio*** | 11.77 ± 2.44 | 4.79 ± 1.94 | N.D. | 0.0058 (**) | A vs B (*) |
| ***Itm2a*** | 1.30 ± 0.38 | 0.29 ± 0.09 | 3.75 ± 1.71 | 0.0005(***) | A vs B (*) B vs C (***) |
| ***Zcchc18*** | 8.24 ± 1.63 | 3.73 ± 0.86 | - 1. ± 1.80 | 0.0031 (**) | A vs B (*) B vs C (**) |
| ***Chst1*** | 535.624 ± 213.70 | 66.53 ± 29.82 | 0.08 ± 0.05 | <0.0001 (***) | A vs C (***) B vs C (**) |
| ***Pim2*** | 0.73 ± 0.19 | 1.11 ± 0.18 | 0.73 ± 0.07 | 0.033 (*) | A vs B (*) |
| ***Rgs16*** | 0.68 ± 0.15 | 0.23 ± 0.09 | - 1. ± 0.15 | 0.0002 (***) | A vs C (*) B vs C (***) |
| ***Met*** | 14.45 ± 4.45 | 27.74 ± 6.64 | 55.71 ± 10.56 | 0.0016 (**) | A vs C (**) |
| ***Bhlh9b*** | 2.50 ± 1.25 | 0.27 ± 0.09 | - 1. ± 0.65 | 0.0002 (***) | A vs C (*) B vs C (***) |
| ***Ifih1*** | 3.48 ± 0.90 | 1.40 ± 0.60 | 0.77 ± 0.22 | 0.0419 (*) | n.s. |
| ***Lag3*** | 10.31 ± 2.20 | 8.35 ± 4.14 | N.D. | 0.347 | n.s. |
| ***Armcx2*** | 1.12 ± 0.86 | 0.14 ± 0.05 | 0.66 ± 0.15 | 0.0146 (*) | B vs C (*) |
| ***Golim4*** | 0.72 ± 0.23 | 0.40 ± 0.17 | - 1. ± 0.36 | 0.001 (**) | A vs C (**) B vs C (***) |
| ***Epbh4*** | 103.05 ± 31.41 | 63.03 ± 21.71 | 55.68 ± 27.56 | 0.541 | n.s. |
| ***Dctd*** | 0.09 ± 0.03 | 0.13 ± 0.03 | 0.96 ± 0.07 | <0.0001 (***) | A vs C (***) B vs C (**) |
| ***Eno2*** | 21.01 ± 14.59 | 18.36 ± 12.57 | N.D. | 0.590 | n.s. |
| ***Pdcd1*** | 7.04 ± 2.72 | 10.06 ± 3.37 | N.D. | 0.381 | n.s. |
| ***Rsad2*** | 20.08 ± 9.16 | 5.44 ± 2.58 | 0.52 ± 0.12 | 0.11 | n.s. |
| ***Cdkn1c*** | 6.03 ± 1.32 | 4.45 ± 0.99 | 3.61 ± 0.65 | 0.724 | n.s. |
| ***Pecam1*** | 4.69 ± 1.13 | 4.40 ± 1.01 | 3.24 ± 0.79 | 0.865 | n.s. |
| ***Xrcc1*** | 12.10 ± 1.97 | 14.59 ± 5.14 | N.D. | 0.974 | n.s. |
|  |  |  |  |  |  |
