# Supplementary material for: Identification of Distinct Unmutated Chronic Lymphocytic Leukemia Subsets in Mice Based on Their T Cell Dependency
Source: Front Immunol. 2018 Sep 13;9:1996. doi: 10.3389/fimmu.2018.01996 (PMC6146083; doi:10.3389/fimmu.2018.01996)
Supplement: Supplementary file 5 [file Table_5.docx]

**Suppl. Table 5 (A):** B-cell receptor characteristics of heterogeneous Human CLL patients.

| **Sample id** | **Gender** | **IGHV** | **IGHD** | **IGHJ** | **Mutational Status** | **CDR3**  **length** | **CDR3** |
| --- | --- | --- | --- | --- | --- | --- | --- |
| KL2015-240 | Female | VH3-30*03 | DH6-13*01 | JH6*02 | 89.73 | 19 | AKVGRPAAFEEYYYYGMDV |
| KL2015-134 | Male | VH4-61*02 | DH5-18*01 | JH4*02 | 90.60 | 12 | ARDPDTYGYVDC |
| KL2017-070 | Male | VH3-30*03 | DH3-09*01 | JH4*02 | 91.03 | 18 | AKPGSVFRYFDWISGLWYW |
| KL2013-025 | Male | VH3-30*03 | DH6-13*01 | JH6*02 | 91.50 | 19 | AKDIYSSSRRVYYDYGMDV |
| KL2014-413 | Male | VH3-30*02 | DH5-12*01 | JH3*02 | 91.90 | 14 | ANELVTSSYDGIDIW |
| KL2013-020B | Male | VH3-30*02 | - | JH6*02 | 91.90 | 19 | ARDGCDSRHCFPFRGWFDP |
| KL2015-241 | Male | VH3-48*03 | DH2-15*01 | JH1*01 | 92.02 | 9 | ARDGGSYPL |
| KL2008-357 | Female | VH4-34*01 | DH1-1*01 | JH6*02 | 92.50 | 20 | ARGYGTTSTTRRYYYYGMDV |
| KL2013-026 | Male | VH3-49*03/05 | DH3-9*01 | JH4*02 | 93.90 | 17 | ARGYYDVLTSYFWDFDY |
| KL2010-250 | Male | VH3-72*01 | DH2-8*01 | JH6*02 | 94.05 | 20 | GRIYCTLSRCSIDQYYGMDV |
| KL2010-510 | Male | VH3-74*01 | DH2-15*01 | JH5*02 | 94.60 | 18 | AREVCIGDNCYSRGWFDP |
| KL2015-555 | Male | VH3-53*01 | DH5-18*01 | JH4*02 | 95.00 | 16 | ARDRGGGYSYGGGFDY |
| KL2013-024 | Female | VH3-7*01 | DH1-26*01 | JH4*02 | 95.50 | 20 | ARVGGFIPKEYSGKWRFFDY |
| KL2013-007 | Male | VH4-30*01 | DH2-2*01 | JH6*03 | 96.02 | 16 | ARDAGVVPVHYYYMDV |
| KL2010-168 | Male | VH3-11*03 | DH2-15*01 | JH4*02 | 97.31 | 13 | ARGGEVMVSPLDR |
| KL2012-317 | Female | VH3-21*01 | DH3-3*01 | JH4*02 | 99.10 | 15 | ATLCCITIFGVSLDY |
| KL2010-451 | Male | VH4-59*01 | DH3-22*01 | JH6*03 | 100.00 | 23 | ARGNYYDSSGYYYVGYYYYYMDV |
| KL2011-447 | Male | VH1-69*01 | DH3-3*01 | JH4*02 | 100.00 | 22 | ARAAVPYYDFWSGYSLDSGFDY |
| KL2011-589 | Male | VH1-69*01 | DH3-3*01 | JH6*03 | 100.00 | 21 | ASGSIFGVVIGSYYYYYYMDVW |
| KL2014-372 | Female | VH1-69*01 | DH3-16*02 | JH5*02 | 100.00 | 22 | ARDPPFDYIWGSYRYRANWFDPW |
| KL2014-420 | Male | VH1-69*01 | DH2-2*01 | JH6*02 | 100.00 | 21 | ASLTIVVVPAAMSYYYYGMDVW |
| KL2015-035 | Male | VH1-69*01 | DH2-2*01 | JH6*02 | 100.00 | 24 | ARDSPHKQDIVVVPAAMVFYSMDV |
| KL2014-260 | Male | VH3-20*01 | DH3-3*01 | JH4*02 | 100.00 | 21 | ARGTGITIFGVVHTTEYYFDYW |
| KL2008-616 | Male | VH3-48*01 | DH3-3*01 | JH6*02 | 100.00 | 21 | ARDCDFWSGYYGYYYYYGMDV |
| KL2016-025 | Male | VH4*31*01 | DH3-22*01 | JH6*02 | 100.00 | 22 | ARDSSPRLYYDSSGYYGMGLDV |
| KL2013-006 | Male | VH4-39*01 | DH3-3*01 | JH6*02 | 100.00 | 26 | ARHASPRDFWSGYPELIYYYYYGMDVW |
| KL2011-399 | Female | VH4-4*02 | DH3-16*02 | JH6*02 | 100.00 | 26 | ARGRRDDYIWGSYRYTDLGYYYGMDV |
| KL2013-083 | Male | VH1-18*01 | DH3-10*01 | JH4*02 | 100.00 | 17 | ARGLQYYGSGSYPYFDY |
| KL2012-169 | Female | VH1-46*01 | DH3-22*01 | JH4*02 | 100.00 | 20 | AREGDSSGYYHVRLVTSFDY |
| KL2013-082 | Male | VH5-51*01 | DH4-23*01 | JH4*02 | 100.00 | 13 | ARQQWLPKENFDY |

**Suppl. Table 5 (B):** B-cell receptor characteristics of stereotypic Human CLL patients.

| **Sample id** | **Gender** | **IGHV** | **IGHD** | **IGHJ** | **Mutational Status** | **Subset#** | **CDR3**  **length** | **CDR3** |
| --- | --- | --- | --- | --- | --- | --- | --- | --- |
| KL2013-424 | Male | VH1-69*01 | DH3-3*01 | JH6*02 | 100.00 | 7H | 26 | CARDFGGNDFWSGYYPNYYYYGMDVW |
| KL2013-305 | Female | VH1-69*06 | DH3-16*02 | JH3*02 | 99.60 | 6 | 23 | CARGGEYDYVWGSYRSNDAFDIW |
| KL2013-384 | Male | VH1-69*01 | DH3-3*01 | JH6*02 | 100.00 | 7H | 26 | CARGVGDYDFWSGYYPNYYYYGMDVW |
| KL2015-426 | Female | VH1-69*01 | DH2-2*01 | JH6*02 | 100.00 | 3 | 24 | CARVVPDIVVVPAAINYYYGMDVW |
| KL2015-467 | Male | VH1-69*01 | DH2-02*02 | JH6*02 | 100.00 | 3 | 24 | CARAVPDIVVVPAALDYYYGMDVW |
| KL2015-367 | Female | VH1-69*01 | DH3-03*01 | JH6*02 | 100.00 | 5 | 22 | CARESGRITIFGSYYYYGMDVW |
| KL2016-485 | Male | VH1-69*01 | DH3-16*02 | JH3*02 | 100.00 | 6 | 23 | CARGGHYDYVWGSYRPNDAFDIW |
| KL2015-442 | Female | VH1-69*06 | DH3-16*02 | JH3*02 | 100.00 | 6 | 23 | CARGGPYDYVWGSYRPPDAFDIW |
| KL2015-420 | Male | VH4-39*01 | DH6-13*01 | JH5*02 | 100.00 | 8 | 21 | CARRKGYSSSWYGRDNWFDPW |
| KL2017-163 | Male | VH4-39*01 | DH6-13*01 | JH5*02 | 100.00 | 8 | 21 | CARRVGYSSSWYSTYNWFDPW |
| KL2006-161 | Female | VH1-3*01 | DH1-7*01 | JH4*02 | 100.00 | 1 | 13 | ARVQALGLPNFDY |
| KL2014-005 | Male | VH5a*01 | DH6-19*01 | JH4*02 | 100.00 | 1 | 13 | ARQQWLGITHFDY |
| KL2016-438 | Male | VH1-08*01 | DH3-10*01 | JH4*02 | 100.00 | 1 | 13 | ARGQWFGEYYFDY |
| KL2009-120 | Male | VH1-3*01 | DH6-19*01 | JH4*02 | 100.00 | 1 | 13 | AREQWLDMPSFDY |
|  |  |  |  |  |  |  |  |  |
