# Supplementary material for: Identification of Distinct Unmutated Chronic Lymphocytic Leukemia Subsets in Mice Based on Their T Cell Dependency
Source: Front Immunol. 2018 Sep 13;9:1996. doi: 10.3389/fimmu.2018.01996 (PMC6146083; doi:10.3389/fimmu.2018.01996)
Supplement: Supplementary file 6 [file Table_6.DOC]

**Suppl. Table 6:** Mean ± SEM expression of indicated genes as measured by qRT-PCR in CLL cells from non-stereotypic U-CLL (n=15), stereotypic U-CLL (#U-CLL, n=14) and M-CLL (n=15) patients. The values indicate relative expression to naïve circulating B cells from healthy volunteers (n=3).

|  | **Unmutated**  **CLL**  **(n=15)** | **# Unmutated**  **CLL**  **(n=14)** | **Mutated**  **CLL**  **(n=15)** | **Kruskal-Wallis**  **Test** | **Dunn’s Multiple comparison**  **test between groups** |
| --- | --- | --- | --- | --- | --- |
|  | **(A)** | **(B)** | **(C)** |  |  |
| ***Chd3*** | 14.74 ± 3.18 | 4.71 ± 1.16 | 9.72 ± 3.88 | 0.015 (*) | A vs B (*) |
| ***Vav3*** | 1.16 ± 0.28 | 0.77 ± 0.20 | 0.65 ± 0.10 | 0.274 | ns |
| ***Clip3*** | 24.08 ± 7.83 | 8.43 ± 3.06 | 3.46 ± 0.84 | 0.025 (*) | A vs C (*) |
| ***Ccdc88a*** | 51.08 ± 11.18 | 61.64 ± 20.00 | 22.63 ± 6.75 | 0.308 | ns |
| ***Trio*** | 0.80 ± 0.18 | 0.75 ± 0.25 | 0.74 ± 0.22 | 0.644 | ns |
| ***Itm2a*** | 10.38 ± 2.88 | 2.14 ± 0.86 | 11.70 ± 4.72 | 0.026 (*) | A vs B (*) |
| ***Zcchc18*** | 13.71 ± 3.83 | 4.23 ± 2.07 | 6.32 ± 1.69 | 0.050 (*) | A vs B (*) |
| ***Pim2*** | 2.18 ± 0.72 | 1.50 ± 0.55 | 0.95 ± 0.26 | 0.104 | ns |
| ***Rgs16*** | 1.63 ± 0.88 | 0.49 ± 0.20 | 3.39 ± 2.18 | 0.330 | ns |
| ***Bhlh9b*** | 7.61 ± 2.20 | 0.40 ± 0.05 | 2.09 ± 0.99 | 0.0001 (***) | A vs B (***) |
| ***Armcx2*** | 3.56 ± 3.56 | 4.72 ± 3.82 | 19.95 ± 10.50 | 0.270 | ns |
| ***Golim4*** | 6.58 ± 1.24 | 3.09 ± 1.56 | 2.36 ± 0.55 | 0.008 (*) | A vs B (**) |
| ***Epbh4*** | 1.15 ± 0.23 | 2.46 ± 1.04 | 0.83 ± 0.13 | 0.4080 | ns |
|  |  |  |  |  |  |
