# Supplementary material for: Identification of Distinct Unmutated Chronic Lymphocytic Leukemia Subsets in Mice Based on Their T Cell Dependency
Source: Front Immunol. 2018 Sep 13;9:1996. doi: 10.3389/fimmu.2018.01996 (PMC6146083; doi:10.3389/fimmu.2018.01996)
Supplement: Supplementary file 7 [file Table_7.DOC]

**Suppl. Table 7 :** (A) List of primers and probes used to test indicated genes in CLL from *IgH.TEμ* mice by real-time quantitative PCR.

| **Gene** | **Forward primer sequence**  **(5’to 3’)** | **Reverse primer sequence**  **(5’to 3’)** | **Universal library probe** |
| --- | --- | --- | --- |
| *Itm2a* | tcgccttcaacaccccta | tctcctgcgggacaactct | #63 (cat. no. 04688627001) |
| *Rgs16* | tgggccagtaagcataacaa | cgtctttaggaaggcatgga | #83 (cat. no. 04689062001) |
| *Ccdc88a* | acgctcagagagaattcagagag | gctgccgtaaggaagacact | #15 (cat. no. 04685148001) |
| *Pim2* | gaggccgaataccgacttg | ttccgggagattactttgatg | #79 (cat. no. 04689020001) |
| *Zcchc18* | aggtggaagccagtttctga | cagaaacctccaaaggaacg | #3 (cat. no. 04685008001) |
| *Vav3* | tgaaggcggagaagtctatga | tgctaggcaacaactccgta | #107 (cat. no. 04692268001) |
| *Chst1* | ctttcaccgccaagtctttc | gctctgccaagccagtatct | #26 (cat. no. 04687574001) |
| *Clip3* | ccaggcaatctcatgctca | ggtcccacagaacctcagc | #94 (cat. no. 04692110001) |
| *Met* | gctgtgctggtcaatggaa | tcagcaccccctatctgc | #79 (cat. no. 04689020001) |
| *Chd3* | agctgaaggagcaaggacac | aggaagtcctccagaaggtctaa | #7 (cat. no. 04685059001) |
| *Armcx2* | cccttcaccctggtcctt | cttcctcgaattaggccaga | #18 (cat. no. 04686918001) |
| *Bhlhb9* | agaatagcatctcccgagca | atgaaaagccacccaactga | #74 (cat. no. 04688970001) |
| *Cdkn1c* | gcaggacgagaatcaagagc | gttctcctgcgcagttctct | #17 (cat. no. 04686900001) |
| *Eno2* | cactaacgtgggggatgaag | gtgtagccagccttgtcgat | #80 (cat. no. 04689038001) |
| *Lag3* | cacctgtagcatccatctgc | ccaggtaacccgaaggattt | #56 (cat. no. 04688538001) |
| *Xrcc1* | gcagaaggaagaggaggaaag | tcctatttggctgagaactcg | #92 (cat. no. 04692098001) |
| *Pdcd1* | ctgcagttgagctggcaat | gcatttgctccctctgaca | #64 (cat. no. 04688635001) |
| *Dctd* | gtgcagtgatgacctgttgc | tcttgttcatgatggcgttc | #25 (cat. no. 04686993001) |
| *Pecam1* | agcaagaagcaggaaggaca | tttcttccatggggcaag | #21 (cat. no. 04686942001) |
| *Ephb4* | tgaatggtgtgtctaccttagcc | caggagaaagaaacccaatgc | #10 (cat. no. 04685091001) |
| *Golim4* | aagcaggtagcccatgaaga | ccacagcattgtaatgagcttg | #68 (cat. no. 04688678001) |
| *Ifih1* | cttgtcacgaacgagatagcc | ccaggacatacgtgctttca | #106 (cat. no. 04692250001) |
| *Trio* | gctctggagcagatagatcacc | tcggcttggttctgatgc | #106 (cat. no. 04692250001) |
| *Rsad2* | cgaggactgcttctgctca | ccaagtattcacccctgtcc | #25 (cat. no. 04686993001) |

**Suppl. Table 6 :** (B) List of primers and probes used to test indicated genes in human CLL by real-time quantitative PCR.

| **Gene** | **Forward primer**  **sequence (5’to 3’)** | **Reverse primer**  **sequence (5’to 3’)** | **Universal library probe** |
| --- | --- | --- | --- |
| *CCDC88A* | acgggatcaagagttcatgc | agtggatgttctgccactatca | #20 (cat. no. 04686934001) |
| *CLIP3* | ctcatgcttagcgcactgg | tccgtggtcccacagaac | #42 (cat. no. 04688015001) |
| *ZCCHC18* | aaaggcgaacgctagtttcc | tggctcagaagcatcactgt | #80 (cat. no. 04689038001) |
| *CHD3* | ttctttgtcaagtgggtaggact | ttcttccgctggtagtttcg | #68 (cat. no. 04688678001) |
| *ITM2A* | gccttcaatacccctaccg | tttttcctgggtggcaact | #63 (cat. no. 04688627001) |
| *RGS16* | ccaccatcctgcctactacg | tccccagacgtgtcttgaa | #60 (cat. no. 04688589001) |
