# Supplementary material for: Identification of Distinct Unmutated Chronic Lymphocytic Leukemia Subsets in Mice Based on Their T Cell Dependency
Source: Front Immunol. 2018 Sep 13;9:1996. doi: 10.3389/fimmu.2018.01996 (PMC6146083; doi:10.3389/fimmu.2018.01996)
Supplement: Supplementary file 8 [file Data_Sheet_1.PDF]

Suppl. Fig. 1: Early onset of disease in V_H11 expressing CLL from *IgH.TEμ* mice

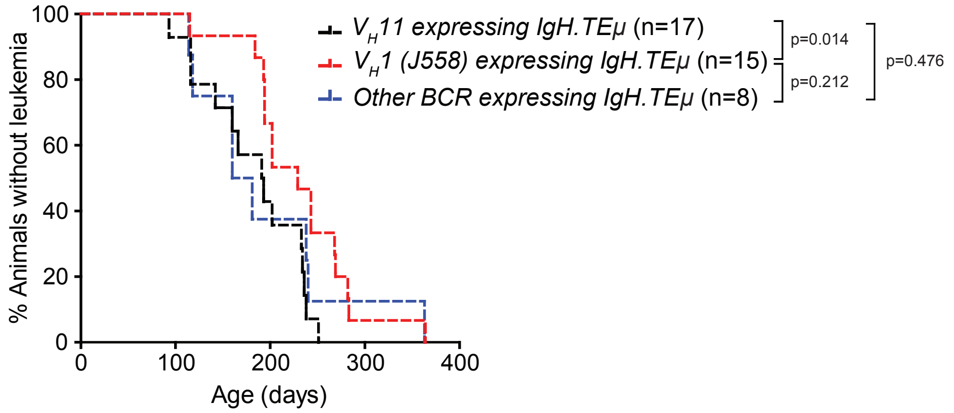

Suppl. Fig. 1: **Early onset of disease in V_H11 expressing CLL from *IgH.TEμ* mice**.

(A) Retrospective Kaplan-Meier incidence curve of CLL expressing a V_H11 (*dotted black line*; n=17), a V_H1 (J558) (*dotted red line*; n=15) or another (non- V_H11/non-V_H1) BCR (*dotted blue line*; n=8) from IgH.TEμ mice. CLL formation was defined by accumulation of >70% IgMb$^+$ B-cells in peripheral blood of the mice. The log rank test was used for calculating the differences in incidence between different mouse groups.

Suppl. Fig.2:
RNA-seq analysis reveals an activated B-cell gene signature in CLL from *IgH.TEμ* mice.

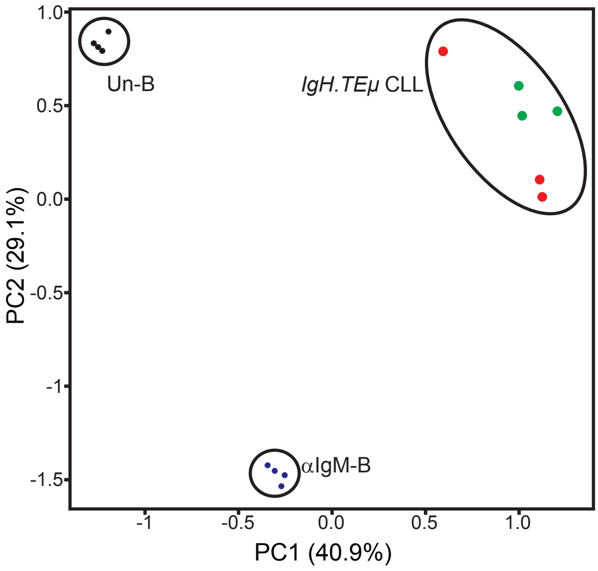

Suppl. Fig. 2: **RNA-seq analysis reveals an activated B-cell gene signature in CLL from *IgH.TEμ* mice.** Principle component analysis (PCA) comparing genome wide expression profiles in unstimulated (n=4, *black*) or anti-IgM-stimulated (n=4, *blue*) WT splenic B cells, $V_H11\text{-}2^+/V_k14\text{-}126^+$ CLL (n=3, *red*) and non-V_H11 (n=3, *green*) BCR expressing tumors from IgH.TEμ CLL mice.

Suppl. Fig. 3: V_H1 CLL represent most heterogenous CLL subgroup in *IgH.TEμ* mice.

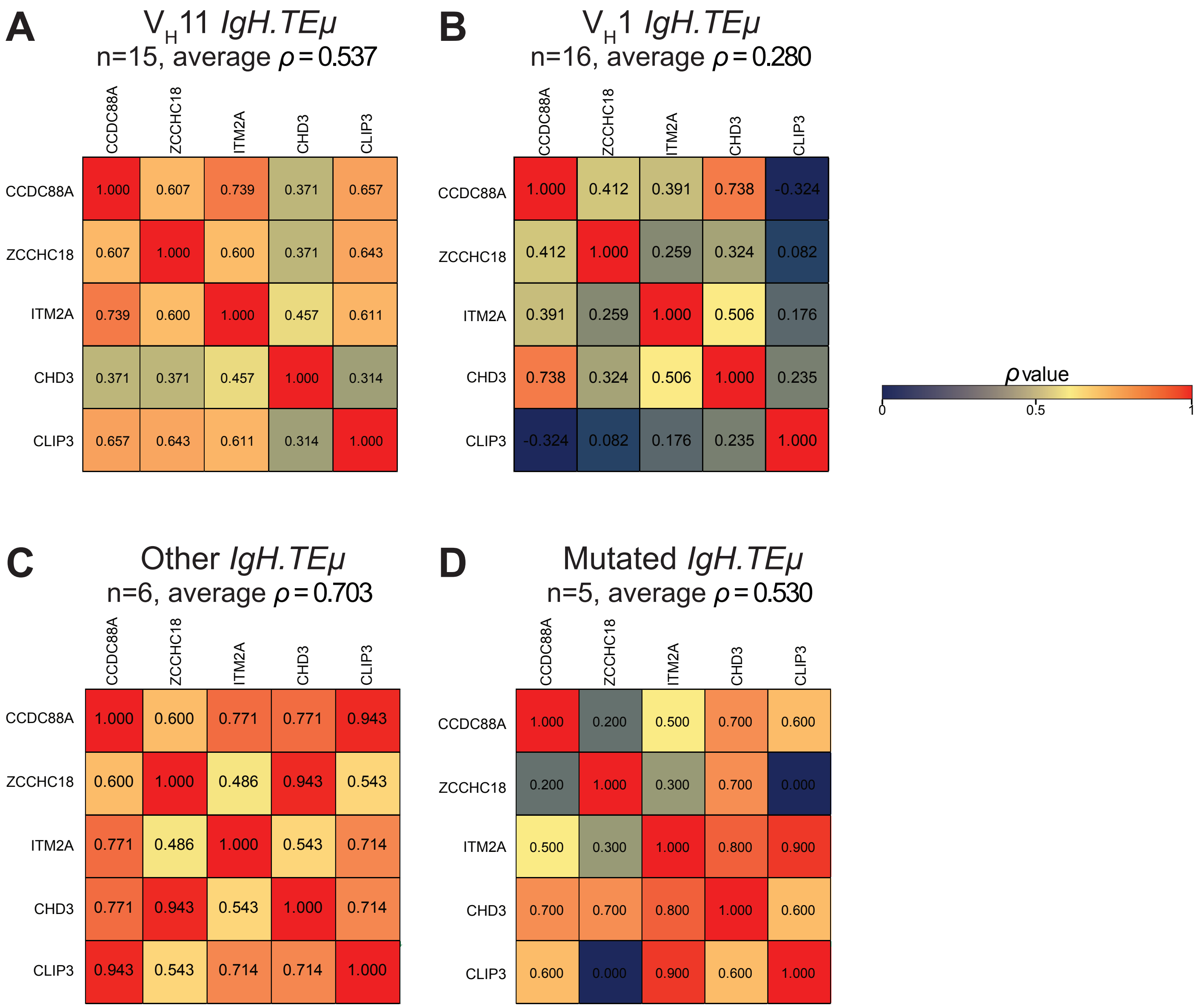

Suppl. Fig. 3: V_H1 CLL represent most heterogenous CLL subgroup in *IgH.TEμ* mice.
Heat map depicting correlation between expression level of indicated genes measured by qRT-PCR for (A) V_H11, (B) V_H1, (C) Others unmutated, and (D) mutated BCR expressing CLL from *IgH.TEμ* mice. Numbers are spearman correlation coefficients (ρ).
